# Landscape of mast cell populations across organs in mice and humans

**DOI:** 10.1084/jem.20230570

**Published:** 2023-07-18

**Authors:** Marie Tauber, Lilian Basso, Jeremy Martin, Luciana Bostan, Marlene Magalhaes Pinto, Guilhem R. Thierry, Raïssa Houmadi, Nadine Serhan, Alexia Loste, Camille Blériot, Jasper B.J. Kamphuis, Mirjana Grujic, Lena Kjellén, Gunnar Pejler, Carle Paul, Xinzhong Dong, Stephen J. Galli, Laurent L. Reber, Florent Ginhoux, Marc Bajenoff, Rebecca Gentek, Nicolas Gaudenzio

**Affiliations:** 1 Toulouse Institute for Infectious and Inflammatory Diseases (Infinity) INSERM UMR1291—CNRS UMR5051—University Toulouse III, Toulouse, France; 2 Centre for Inflammation Research and Centre for Reproductive Health, Queens Medical Research Institute, University of Edinburgh, Edinburgh, UK; 3 Aix Marseille University, CNRS, INSERM, Centre d'immunologie de Marseille-Luminy, Marseille, France; 4 Institut Necker des Enfants Malades, CNRS UMR8253, Paris, France; 5 Department of Medical Biochemistry and Microbiology, Uppsala University, Uppsala, Sweden; 6 Toulouse University and Centre Hospitalier Universitaire, Toulouse, France; 7 The Solomon H. Snyder Department of Neuroscience, School of Medicine, Center for Sensory Biology, Johns Hopkins University, Baltimore, MD, USA; 8 Howard Hughes Medical Institute, Johns Hopkins University School of Medicine, Baltimore, MD, USA; 9 Departments of Pathology and Microbiology and Immunology, Stanford University, Stanford, CA, USA; 10 Sean N. Parker Center for Allergy and Asthma Research, Stanford University, Stanford, CA, USA; 11 Singapore Immunology Network , Agency for Science, Technology and Research, Singapore, Singapore; 12 Gustave Roussy Cancer Campus, Villejuif, France; 13 INSERM U1015, Gustave Roussy, Villejuif, France; 14 Shanghai Institute of Immunology, Shanghai JiaoTong University School of Medicine, Shanghai, China; 15 Translational Immunology Institute, SingHealth Duke-NUS Academic Medical Centre, Singapore, Singapore; 16 Genoskin SAS, Toulouse, France

## Abstract

Mast cells (MCs) are tissue-resident immune cells that exhibit homeostatic and neuron-associated functions. Here, we combined whole-tissue imaging and single-cell RNA sequencing datasets to generate a pan-organ analysis of MCs in mice and humans at steady state. In mice, we identify two mutually exclusive MC populations, MrgprB2^+^ connective tissue–type MCs and MrgprB2^neg^ mucosal-type MCs, with specific transcriptomic core signatures. While MrgprB2^+^ MCs develop in utero independently of the bone marrow, MrgprB2^neg^ MCs develop after birth and are renewed by bone marrow progenitors. In humans, we unbiasedly identify six MC clusters/states (MC1–6) distributed across 12 organs with different transcriptomic core signatures. MC1 are preferentially enriched in the skin and lungs, MC2, MC3, and MC4 in the skin and bladder, MC5 in the lymph node and vasculature, and MC6 in the trachea and lungs. This comprehensive analysis offers valuable insights into the natural diversity of MC subtypes in both mice and humans.

## Teaser

Combining whole-tissue imaging and single-cell RNA sequencing datasets, Tauber et al. present a pan-organ analysis of mast cells in mice and humans at steady state, revealing an unexpected heterogeneity of mast cell populations across tissues and species.

## Graphical Abstract

**Figure:**
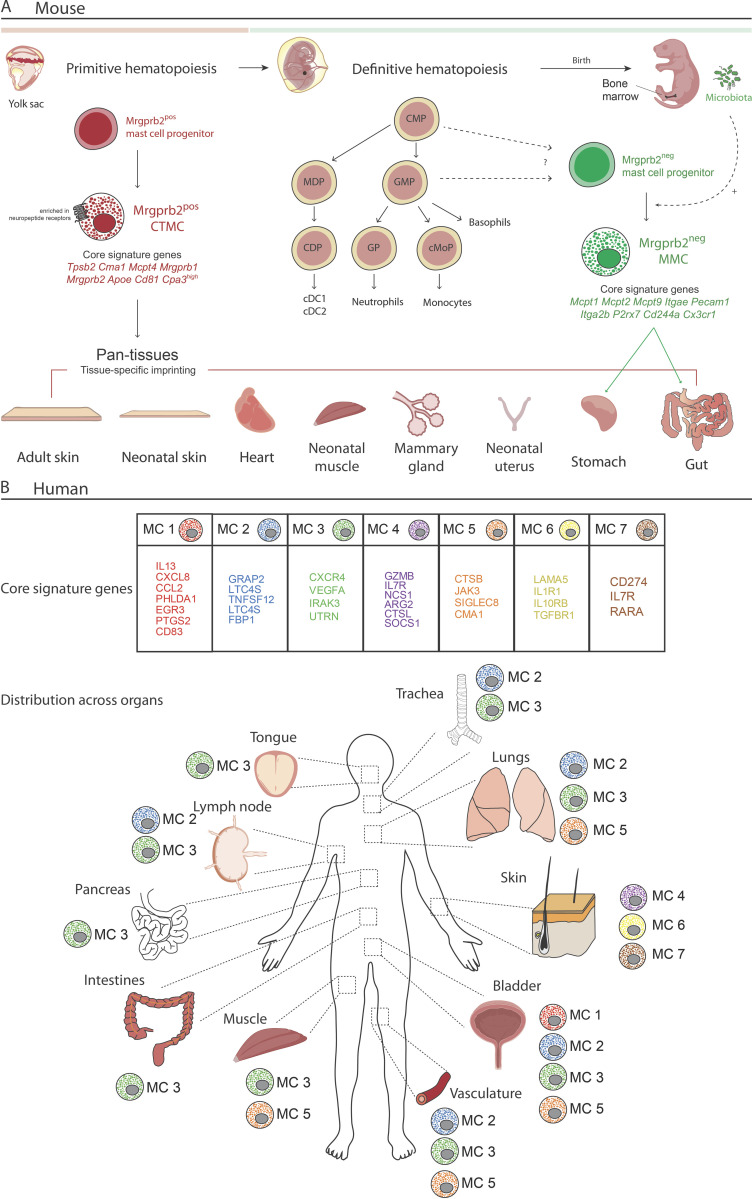

## Introduction

Mast cells (MCs) are distributed in virtually all organs and have been described as important cellular players in many pathological contexts, including allergy (Galli et al., 2020a, 2008; Gurish and Austen, 2012; Kim et al., 2020). However, they also can be beneficial during infections (Abraham and St. John, 2010; Boyce, 2020; Galli et al., 2020a; Marshall, 2004) and as a source of venom detoxification factors (Galli et al., 2020b; Marichal et al., 2013; Metz et al., 2006; Palm et al., 2013).

MCs are thought to be of mixed embryonic origins, arising from both yolk sac– and hematopoietic stem cell (HSC)–derived progenitors (Gentek et al., 2018; Li et al., 2018; Nilsson and Dahlin, 2019), and it has been assumed that embryonic MCs are then slowly replaced by bone marrow (BM)–derived progenitors in the adult mouse. Histochemical analyses have classified MCs into two main categories, mucosal MCs (MMCs) and connective tissue–type MCs (CTMCs; Befus et al., 1985). MMCs are found mostly in the mucosa of the gut and lungs, and CTMCs are found in the skin and the peritoneal cavity (Katz et al., 1985). While such approaches have enabled many important discoveries, one of the greatest challenges in the field of MC biology is to define the full heterogeneity of MC populations across organs and to understand whether multiple tissue niches are associated with microenvironment-specific MC functions. The use of single-cell RNA sequencing (scRNAseq) technologies provides a great opportunity to perform a comprehensive analysis of tissue-resident MCs (Cildir et al., 2021), and a recent report has shown that MCs infiltrating the airway mucosa in patients with type 2 disorder could exhibit CTMCs-like and MMCs-like phenotypes, but also one proliferating and one intermediate phenotype with a distinct transcriptomic effector program (Dwyer et al., 2021). These findings strongly suggest that MCs’ classification might be extended beyond the classical CTMC/MMC dichotomy.

Bilateral interactions between the immune and nervous systems have emerged as critical for the maintenance of tissue homeostasis (Basso et al., 2019; Veiga-Fernandes and Artis, 2018). We and others have recently shown that CTMCs expressing the Mas-related G protein–coupled receptor B2 (MrgprB2, the mouse ortholog to MRGPRX2; Ali, 2021; McNeil et al., 2015; Roy et al., 2021) are associated with nociceptors in the skin and communicate with such neurons (Meixiong et al., 2020) to regulate pain, itch, and type 2 inflammation (Green et al., 2019; Meixiong et al., 2019; Serhan et al., 2019). However, the ontogeny and functional heterogeneity of MCs across tissues are poorly defined and remain a promising area of exploration to improve both MC annotation and understanding of their specific functions in mice and humans.

Here, we integrate multiple single-cell datasets from in-house and publicly available sources (all decontaminated from potential ambient tissue mRNA) to build a comprehensive overview of mouse and human MC populations across organs. In the mouse, CTMCs expressing MrgprB2^+^ have a common gene expression program across organs, such as in skin, muscle, uterus, mammary gland, peritoneal cavity, and heart. By contrast, MrgprB2^neg^ MMCs are transcriptionally distinct from MrgprB2^+^ CTMCs and are mostly found in the digestive tract. While MrgprB2^+^ CTMCs develop during embryogenesis and are independent of the BM for renewal, MrgprB2^neg^ MMCs arise postnatally, require signals from the microbiota, and are renewed by Ms4a3^neg^ BM progenitors. We found that MrgprB2^+^, but not MrgprB2^neg^, MCs are required for food-induced systemic anaphylaxis. In humans, the unbiased analysis of all MCs identified across 24 organs in the human cell atlas revealed the presence of six distinct populations/states, named MC1–6, with specific groups of genes allowing their precise identification. We show that the six MC clusters can be found heterogeneously distributed across the skin, lung, pancreas, skeletal muscle, tongue, bladder, large and small intestines, lymph nodes, mammary glands, trachea, and vasculature, with nevertheless organ-specific enrichments per clusters.

This study provides a general framework to decipher MCs’ heterogeneity across organs in mice and humans via the generation of an online transcriptomic resource that regroups multiple MC transcriptomic profiles. Such an MC single-cell compilation should help to standardize MC annotation and better understand specialized MC functions in mice and humans.

## Results

### Single-cell profiling of mouse CTMCs and MMCs reveals two distinct transcriptomic programs

**Figure 1.**
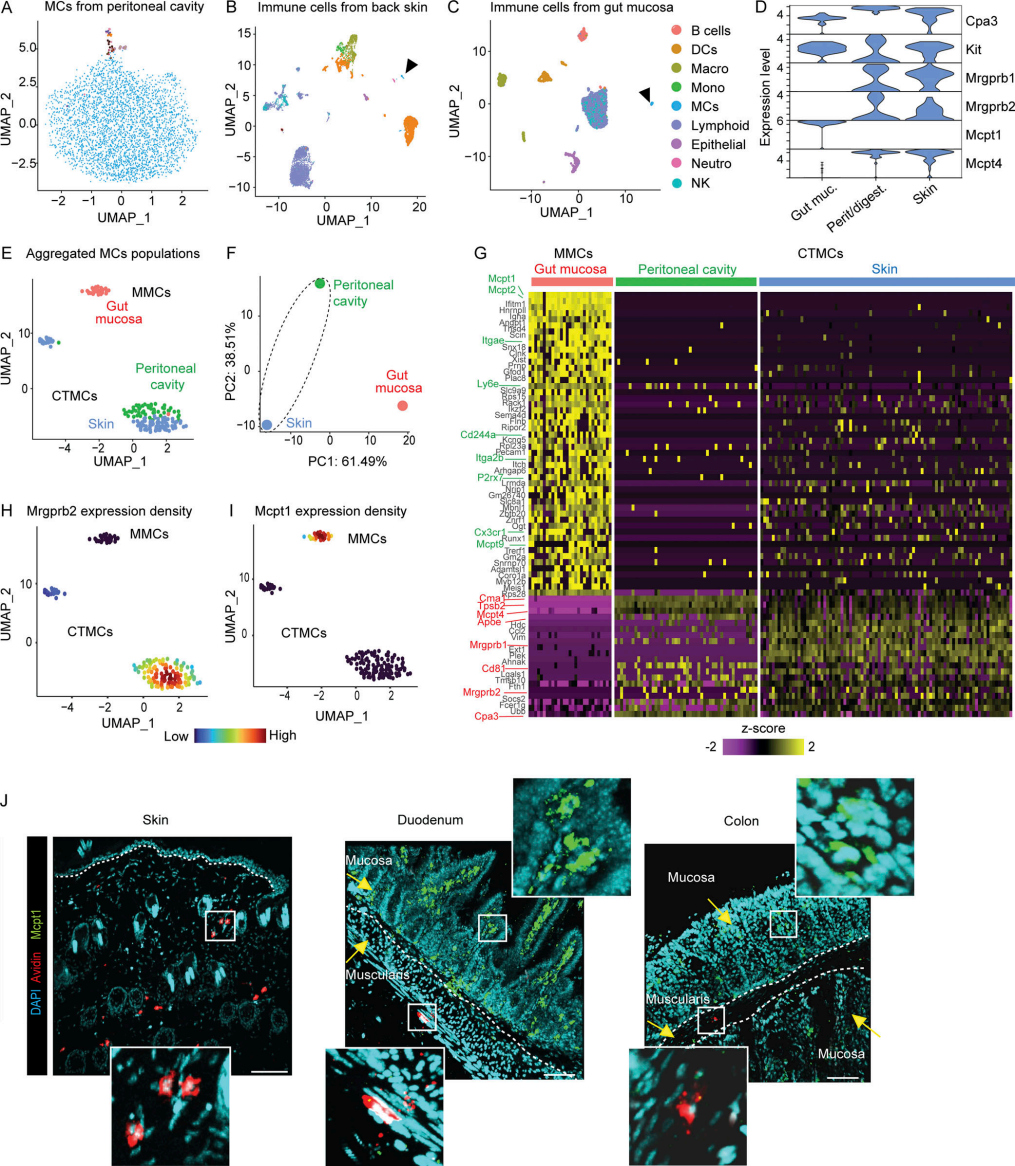
***Mrgprb2***^***+***^ **and *Mrgprb2***^***neg***^ **MCs represent transcriptionally distinct MC populations. (A)** UMAP plot of the scRNAseq performed on sorted peritoneal cavity. MCs. **(B)** UMAP plot of the scRNAseq performed on sorted immune cells from the back skin. Black arrowhead: MC population. **(C)**. UMAP plot of the scRNAseq performed on sorted immune cells from the gut mucosa. Black arrowhead: MC population. **(D)** Violin plot of the expression of *Mrgprb2*, *Mrgprb1*, *Mcpt4*, *Mcpt1*, *Kit*, and *Cpa3*. **(E)** UMAP plot of all MC populations aggregated. **(F)** PCA showing the segregation between skin/peritoneal cavity and gut MCs. **(G)** Heatmap of 74 representative DEGs between MCs from gut, peritoneal cavity, and skin MC related. Characteristic genes and surface markers upregulated in gut (green) or peritoneal cavity and skin MCs (red) are highlighted. **(H and I)** (H) *Mrgprb2* and (I) *Mcpt1* expression density on the aggregated populations. **(J)** Representative 3D confocal microscopy images of Avidin SRho (red), Mcpt1 (green), and DAPI (cyan) fluorescent signals of back skin, duodenum, and colon. Arrows indicate gut mucosa and muscularis. Data are representative of three independent experiments. Bars = 100 (skin) and 70 (duodenum and colon) μm. DC, dendritic cell; NK, natural killer.

We first isolated CD45^+^ CD117^+^ MCs from the peritoneal cavity (which includes cells free in the peritoneum and populations attached to the mesentery and the outer parts of the digestive tract) as a well-defined MC population and CD45^+^ immune cells from both the skin and the gut mucosa (two anatomical sites enriched in CTMCs and MMCs, respectively) of WT C57BL/6J mice by FACS. We used 10X scRNAseq to generate transcriptional profiles for each individual cell (quality controls are described in the Materials and methods section). As droplet-based single-cell technologies can suffer from the presence of cross-contamination from ambient mRNA in each droplet, we pretreated all datasets with Decontx (Yang et al., 2020), a Bayesian method that calculates and removes potential contamination in individual cells (the detailed protocol is described in the Materials and methods section). Using the Uniform Manifold Approximation and Projection (UMAP) approach, the expression patterns of 12,792 detected genes displayed one unique MC reference cluster in the peritoneal cavity representing ∼99% of all sorted cells (Fig. 1 A). In the skin and the gut mucosa, we analyzed the expression patterns of 22,606 and 18,913 genes, respectively, and could unambiguously identify a relatively homogenous MC cluster in each tissue (Fig. 1, B and C). All MC populations were annotated based on their combined expression of two cardinal MC genes: *Cpa3* and *Kit*. Interestingly, two genes encoding (neuro) receptors of the Mrg family, *Mrgprb2* and *Mrgprb1*, and the gene *Mcpt4* were highly expressed in the populations of MCs from the peritoneal cavity and the skin, while gut mucosa-associated MCs selectively expressed *Mcpt1* (a well-known marker for MMCs; Fig. 1 D). When the three MC datasets were integrated together on a UMAP or using the principal component analysis (PCA) approach to obtain a high-level view of CTMC and MMC populations, skin and peritoneal cavity MCs aggregated in two narrow clusters, while gut mucosal MCs segregated separately (Fig. 1, E and F).

We next assessed differentially expressed genes (DEGs) among our MC datasets. We found 195 DEGs (adjusted P value <0.05) between gut MMCs and the clusters formed by skin and peritoneal cavity CTMCs, confirming the presence of two independent MC populations with intrinsic transcriptomic core signatures (Fig. 1 G and Table S1). Among the most significant DEGs, we notably found high expression of genes in gut MMCs encoding MC proteases (*Mcpt1*, *Mcpt2*, and *Mcpt9*) and surface proteins of the integrin family (*Itgae* and *Itga2a*), a purinoceptor (*P2rx7*), the lymphocyte antigen six family member E (*Ly6e*), the platelet endothelial cell adhesion molecule 1 (*Pecam1*, also known as CD31), and a chemokine receptor (*Cx3cr1*). Conversely, the other clusters of CTMCs were found enriched in genes notably encoding other MC proteases (*Cma1*, *Mcpt4*, *Tpsb2*, and *Cpa3*), two receptors of the Mrg family (*Mrgprb2* and *Mrgprb1*), genes of fat metabolism (*Apoe*), chemokine (*Ccl2*), and tetraspanin family (*Cd81*; Fig. 1 G). Using the aggregated UMAP of all MCs, we could selectively identify two MC populations based on the expression of *Mrgprb2*, with a large proportion of skin and peritoneal cavity CTMCs being *Mrgprb2* positive and gut MMCs being *Mrgprb*2 negative (and *Mcpt1* positive; Fig. 1, H and I).

**Figure S1.**
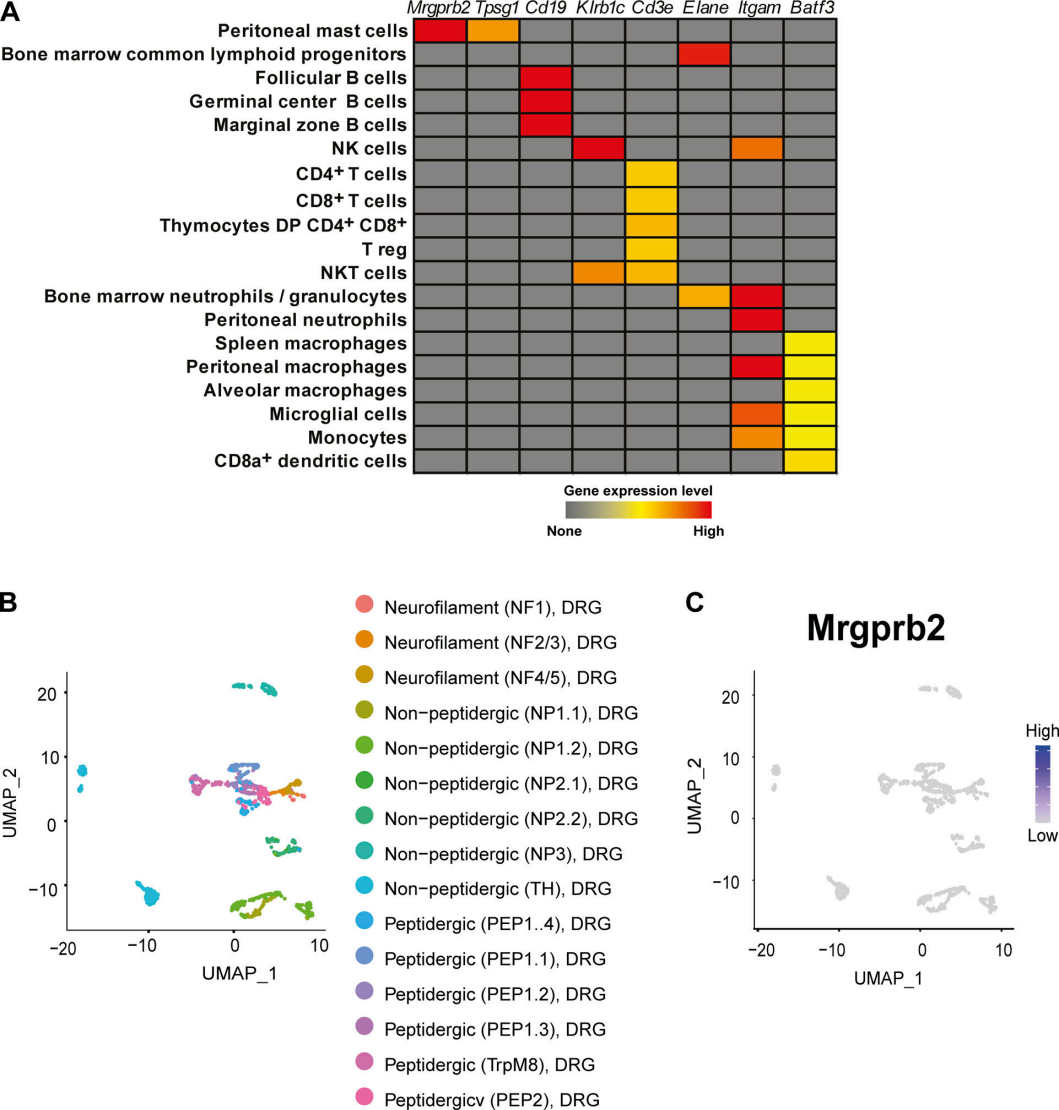
**MrgprB2 expression is restricted to MCs in the mouse. (A)** Publicly available microarray gene expression data (Immunological Genome Project) of *Mrgprb2* in different mouse immune cells; data are shown using a heat map of mRNA expression levels. The expression of the genes *Tpsg1*, *Cd19*, *Klrb1c*, *Cd3e*, *Elane*, *Itgam*, and *Batf3* is presented as reference. **(B)** UMAP projection of the DRG neuron populations identified in Zeissel et al. (2018). **(C)** UMAP projection of the expression of MrgprB2 in the DRG neurons. NK, natural killer.

**Figure S2.**
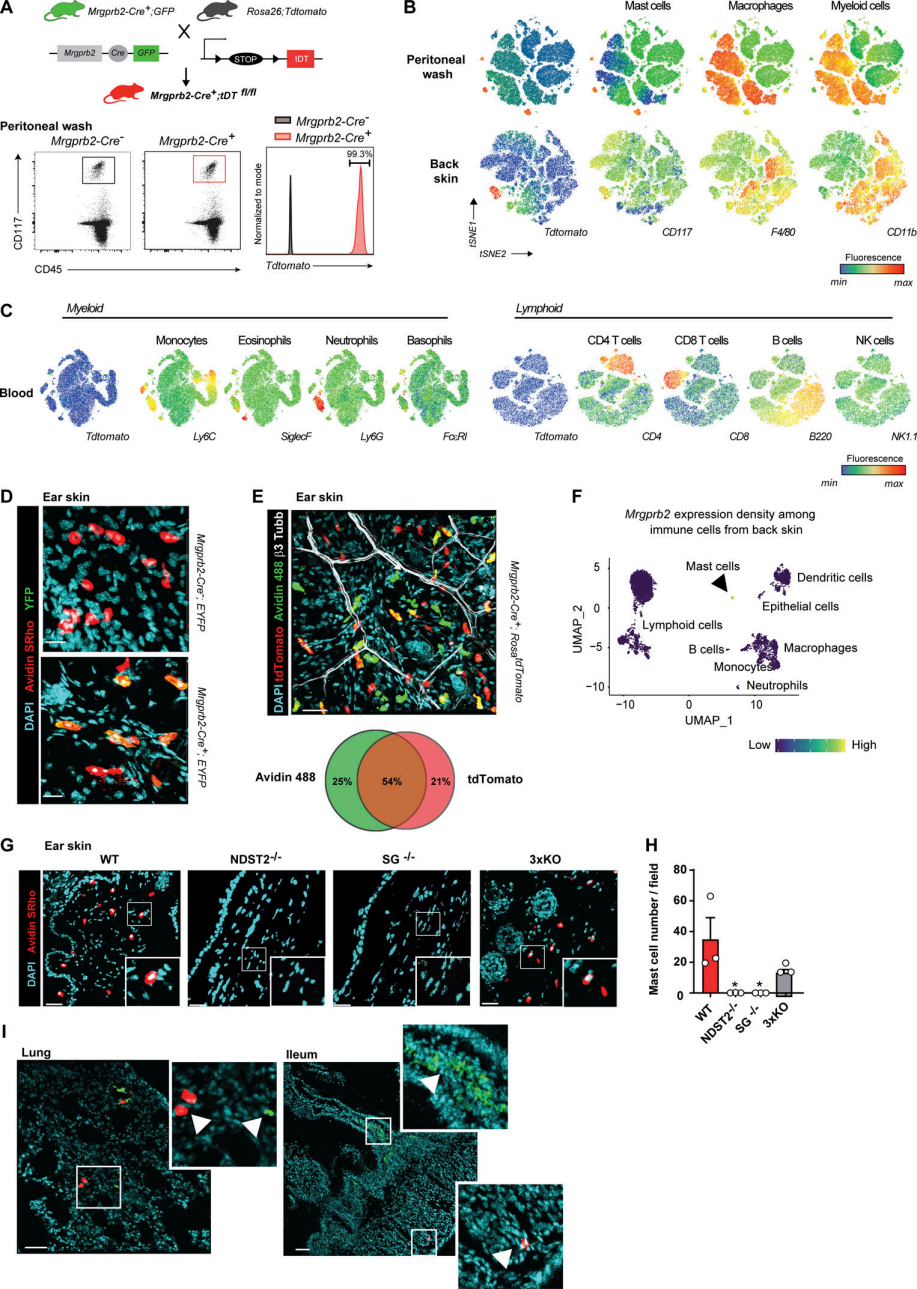
**MrgprB2-cre mice allow the tracing of MCs across tissues. (A)** Protocol used to selectively trace MrgprB2^+^ MCs in 6–8-wk-old mice. Peritoneal cells were isolated from *Mrgprb2-Cre*^*+*^*; Rosa*^*tdTomato*^ or *Rosa*^*tdTomato*^ control mice, and MCs were analyzed by flow cytometry based on CD117 expression. **(B–D)** tdT fluorescence was assessed in *Mrgprb2-Cre*^*+*^*; Rosa*^*tdTomato*^ (red curve) as compared with *Rosa26;tdTomato* (gray curve). **(B)** t-SNE of the tdT, CD117, F4/80, and CD11b expression in CD45^+^ immune cells from peritoneal lavage (upper row) and back skin (lower row) in *Mrgprb2-Cre*^*+*^*; Rosa*^*tdTomato*^ mice. **(C)** t-SNE of the tdT, Ly6c, SiglecF, Ly6g, FcεRI, CD4, CD8, B220, and NK1.1 expression in CD45^+^ immune cells from blood in *Mrgprb2-Cre*^*+*^*; Rosa*^*tdTomato*^ mice. **(D)** Representative 3D confocal microscopy images of Avidin (Av.SRho, red) and EYFP (green) fluorescent signals of ear skin sections from *Mrgprb2-Cre*^*+*^*; EYFP* mice, compared to littermate controls. **(E)** Representative confocal microscopy image of skin from *Mrgprb2-Cre*^*+*^*; Rosa*^*tdTomato*^ mice (red) stained with Avidin 488 (green) and β3-Tubulin (white; upper panel), and Venn diagram showing the colocalization between Av^+^ and Tdtomato^+^ cells (number of cells are shown in the circles). **(F)** UMAP representation of *Mrgprb2* expression density among immune cells from back skin. **(G)** Representative confocal microscopy images of Avidin (Av.SRho, red) fluorescent signal in skin sections of WT mice and mice deficient for serglycin (SG), N-deacetylase/N-sulfotransferase 2 (NDST2) or three major MC proteases (3xKO: Mcpt4, Mcpt6, and Cpa3). **(H)** MCs numbers in skin sections of WT (red bar), SG^*−/−*^ (gray bars), *NSTD2*^*−/−*^ (gray bars), or *Mcpt4*^*−/−*^*; Mcpt6*^*−/−*^*; Cpa3*^*−/−*^ (3xKO, gray bars) mice. Each circle = one mouse. **(I)** Representative confocal microscopy image of lung and ileum from *Mrgprb2-Cre*^*+*^*; Rosa*^*tdTomato*^ mice (red) stained with Mcpt-1 (green). Arrowheads indicate individual MCs. Scale bars = 20 µm (D) and 50 µm (E, G, and I). Number of mice: A–C, *n* = 4 mice, one experiment; D, *n* = 3 per group two experiments; F and G, *n* = 3 per group, one experiment; mean ± SEM; one-way ANOVA with Tukey’s test for multiple comparisons (H), *P < 0.05.

Using the Immgen database (https://www.immgen.org/) and publicly available single-cell datasets (Zeisel et al., 2018) from mouse dorsal root ganglia (DRG), we confirmed that *Mrgprb2* expression was restricted to MCs among all the analyzed immune cells (Fig. S1 A) and sensory neurons (Fig. S1, B and C) in the mouse. We next used *Mrgprb2-Cre;Rosa*^*Tdtomato*^ mice (McNeil et al., 2015) to trace *Mrgprb2*-expressing cells in different immune compartments by flow cytometry. MCs from both peritoneal lavage (Fig. S2 A) and skin expressed the TdTomato (Tdt), while all analyzed immune cells from the peritoneal wash, skin, and blood, including basophils, did not display any detectable Tdt signal (Fig. S2, B and C). These data confirmed that *Mrgprb2* expression is certainly restricted to CTMCs at steady state, at least to those MCs found in the skin and peritoneal cavity.

Previous studies reported that skin MCs could be efficiently stained with fluorescent avidin molecules (Gaudenzio et al., 2016; Reber et al., 2017; Schäfer et al., 2013; Serhan et al., 2019). We used both *Mrgprb2-Cre*^*+*^*; EYFP* and *Mrgprb2-Cre*^*+*^*; Rosa*^*Tdtomato*^ mice, stained their skin with sulforhodamine (Av.SRho)- or Alexa488 (A488)-labeled avidin, and confirmed that EYFP and Av.SRho or Tdt and Av.A488 fluorescent signals colocalized in a large proportion (54%) of β3-tubulin^+^ neuronal fibers near skin MCs (Fig. S2, D and E). We observed the presence of some Avidin^+^ MCs that were not positive (or very weakly positive) for the Tdt (25%) and also some Tdt^+^ cells that were not positive (or very weakly positive) for Avidin (21%; Fig. S2 E). Previous reports have suggested that virtually all *Mrgprb2*^*+*^ MCs in the hairy and glabrous skin were also Avidin^+^ (McNeil et al., 2015). This slight discrepancy could be explained, at least in part, by the protocols used to stain all *Mrgprb2*^*+*^ MCs with Avidin in different organs or the capacity to detect the tracer Tdt and/or differential expression of *Mrgprb2* depending on the animal care facility environment. However, when analyzed by scRNAseq on CD45^+^ skin immune cells, we confirmed that *Mrgprb2* expression was entirely restricted to MCs (Fig. S2 F). Using mice deficient for either serglycin, heparan sulfate N-deacetylase/N-sulfotransferase 2, or all three major MC proteases (i.e., Mcpt4, Mcpt6, and Cpa3; Galli et al., 2015; Wernersson and Pejler, 2014), we next found that Av.SRho can be used to identify the sulfated form of heparin/heparan sulfate in the granules of a large proportion of CTMCs in the skin (Fig. S2, G and H).

Our scRNAseq analysis (Fig. 1, H and I) suggested that the expression of *Mrgprb2* and *Mcpt1* was mutually exclusive in the two different MC populations. We thus hypothesized that staining with Av.SRho or anti-Mcpt1 antibody could be used as selective markers of MrgprB2^+^ CTMC (identified as Av.SRho^+^ Mcpt1^neg^) and MrgprB2^neg^ (identified as Av.SRho^neg^ Mcpt1^+^) MMC populations in different organs. Indeed, we found that skin MCs were exclusively composed of Av.SRho^+^ Mcpt1^neg^ CTMCs (Fig. 1 J). In different segments of the gastrointestinal (GI) tract, we found that the two populations of MCs coexist (Fig. 1 J), however, nested in specific anatomical niches. Av.SRho^neg^ Mcpt1^+^ MMCs were very abundant and found exclusively in the lamina propria in all intestinal segments analyzed. Conversely, Av.SRho^+^ Mcpt1^neg^ gut CTMCs were very rare and resided within the muscularis propria. We could confirm the presence of both *Mrgprb2*^*+*^ MCs and *Mcpt-1*^*+*^ MCs in the lungs and GI tract of Mrgprb2-reporter mice (Fig. S2 I). These data are in line with previous observations, based on classical histochemistry (de Lisle et al., 2009), of the presence of CTMCs in the gut muscularis. Overall, these results confirm that MrgprB2^+^ CTMCs and MrgprB2^neg^ MMCs are two different MC subsets that can be selectively identified based on either their transcriptomic profile or their mutually exclusive staining with Av.SRho and anti-Mcpt1 antibody.

### MrgprB2^+^ and MrgprB2^−^ MCs are independent MC subsets conserved across organs in mice

**Figure 2.**
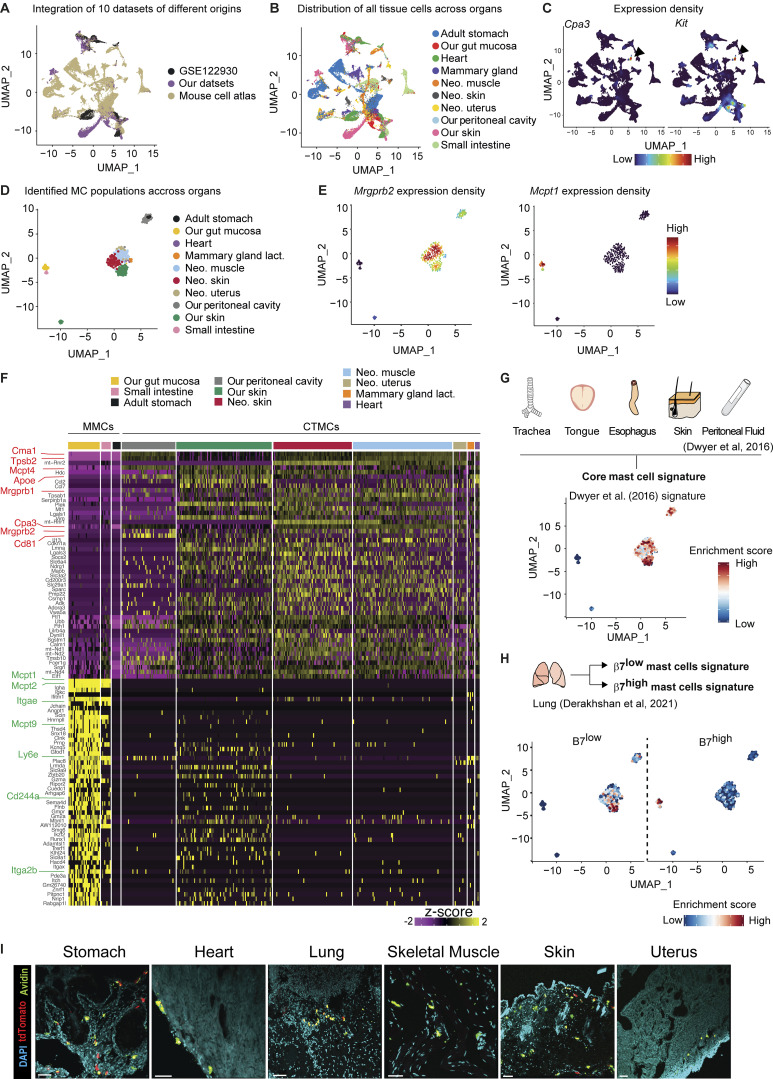
**Transcriptomic profiling of mouse MCs across tissues. (A)** UMAP plot showing the origin of the dataset used for MC identification. **(B)** UMAP of the distribution of all tissue cells across organs. **(C)** *Cpa3* (left) and *Kit* (right) expression density on the aggregated dataset. Black arrowhead: MC population. **(D)** UMAP of integrated MCs according to their tissue of origin. **(E)** *Mrgprb2* and *Mcpt1* expression density on the aggregated populations. **(F)** Heatmap of 85 representative DEGs between MMC and CTMC populations. Characteristic genes and surface markers upregulated in *Mrgprb2*^*−*^ (green, GI tract) or *Mrgprb2*^*+*^ MC (red, other organs) are highlighted. **(G)** UMAP showing the score-based identification of the common transcriptomic signature of MCs from Dwyer et al. (2016). **(H)** UMAP showing the score-based identification of the β7^low^ and β7^high^ MCs from Derakhshan et al. (2021). **(I)** Representative 3D confocal microscopy images of Avidin 488 (green) Tdt^+^ (red) MCs in stomach, heart, lung, skeletal muscle, back skin, and uterus of MrgprB2-reporter mice. Data are representative of two independent experiments with at least two animals per group. Scale bars = 50 µm. Neo., neonatal.

To better understand MC heterogeneity across tissues, we took advantage of the large single-cell database of the Mouse Cell Atlas (http://bis.zju.edu.cn/MCA/atlas.html) and of a recent publicly available resource to integrate datasets from 10 organs together (Fig. 2, A and B). We first pretreated our datasets with Decontx (Yang et al., 2020) and then extracted the gene signature of MC populations based on their co-expression of the two genes *Cpa3* and *Kit* (Fig. 2 C)*.* We could identify clear MC signatures in the small intestine, stomach, uterus, mammary gland, neonatal skin, skeletal muscle, and heart. We next integrated all the datasets, as described in the Materials and methods section, and projected these scRNAseq datasets together with our datasets (Fig. 1) on the same UMAP (Fig. 2 D) and reached a total number of 382 single MCs analyzed from multiple organs. We could again identify two global clusters based on a UMAP representation, one large cluster composed of *Mrgprb2*^+^ CTMCs found in the skin, peritoneal cavity, skeletal muscle, mammary gland, uterus, stomach, and heart, and another one composed of *Mrgprb2*^−^ (*Mcpt1*^*+*^) MMCs from global gut mucosa and small intestine (Fig. 2, D and E). Here again, we found the same top DEGs distinguishing CTMC and MMC populations (albeit more DEGs were found, i.e., 330 vs. 195, in Fig. 1 E), and a relatively conserved signature of certain genes was seen among all the *Mrgprb2*^+^ CTMC populations from the different tissues (Fig. 2 F and Table S2). We next projected on the UMAP the previously published common microarray-based signature of MCs sorted from trachea, esophagus, skin, and peritoneal wash reported by Dwyer et al. (2016) and of lung MCs identified by Derakhshan et al. (2021). We found that our CTMC populations exhibited a conserved transcriptomic profile with both the common signature of MCs described by Dwyer et al. (2016) (Fig. 2 G) and the β7^low^ MC population identified by Derakhshan et al. (2021) (Fig. 2 H). Conversely, our MMC populations shared a common transcriptomic signature with the β7^high^ MC population identified by Derakhshan et al. (2021) in the lung (Fig. 2 H). Finally, using Mrgprb2-reporter mice, we could confirm the presence of *Mrgprb2*^*+*^ MCs in all tissues in which they were detected by scRNAseq (Fig. 2 I). These results indicate that *Mrgprb2*-expressing CTMCs are not restricted to the skin and peritoneal cavity but instead represent an MC population that is distributed across different tissues in the mouse with a conserved transcriptomic core, being significantly distinct from that of the *Mrgprb2*-negative MMCs found in the intestinal tract.

### In the skin, *Mrgprb2*^low^ and *Mrgprb2*^high^ CTMCs co-exist and have different maturation state

**Figure S3.**
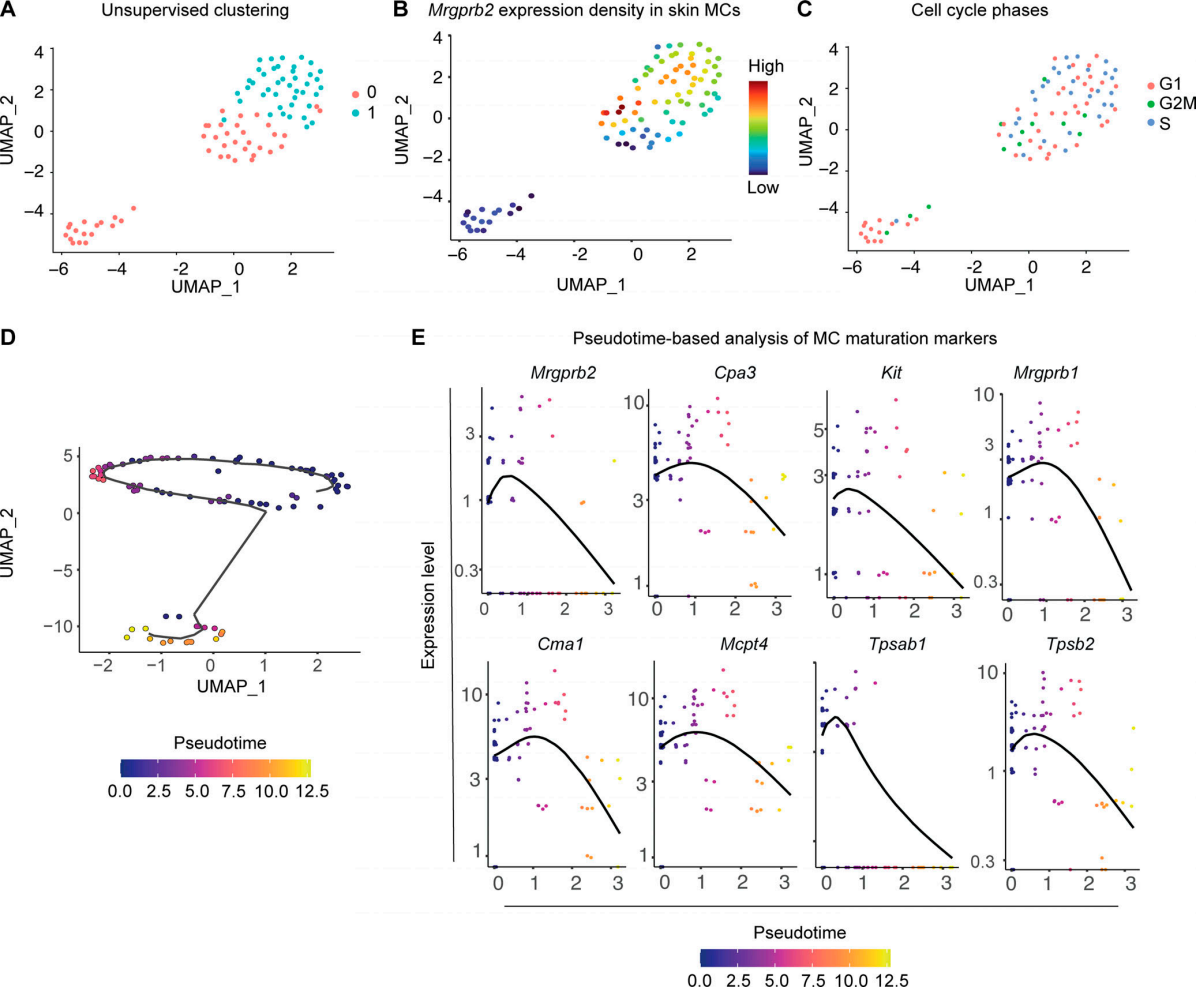
**MrgprB2**^**high**^ **MCs in the skin represent a mature population of MCs in the mouse. (A and B)** UMAP showing (A) the unsupervised Louvain clustering and (B) *Mrgprb2* expression density of isolated skin MCs. **(C)** UMAP showing the cell cycle state of skin MCs. **(D)** Monocle analysis of developmental trajectories in isolated skin MCs. **(E)** Single-cell expression of *Mrgprb2*, *Cpa3*, *Kit*, *Mrgprb1*, *Cma1*, *Mcpt4*, *Tpsab1*, and *Tpsb2* mRNA along the pseudotime scale.

When analyzing the density of *Mrgprb2* expression in our skin MC dataset, we observed the presence of a major group of *Mrgprb2*^high^ MCs and a smaller group of *Mrgprb2*^low^ MCs (Fig. 1, E–H; and Fig. 2, D and E). We thus investigated whether these two groups were transcriptionally distinct populations or whether they might represent the same population but at a different maturation state. When we isolated our skin MCs and applied a classical unsupervised analysis, we could find two clusters (Fig. S3 A), but that did not segregate according to Mrgprb2 expression density (Fig. S3 B), strongly suggesting that *Mrgprb2*^high^ and *Mrgprb2*^low^ MCs might belong to the same population of skin CTMCs. We next wondered whether differences in proliferative state could underlie the clusters identified by unsupervised analysis. However, no difference in cell cycle phases between the two groups of MCs was identified (Fig. S3 C). We next inferred a potential differentiation trajectory using *Monocle3* (Cao et al., 2019; Fig. S3, D and E). This analysis could identify an expression gradient along the pseudotime axis of key MC maturation genes including *Cma1*, *Mcpt4*, *Tpsab1*, and *Tpsb2* associated with the level of expression of both *Mrgprb2* and *Mrgprb1*, suggesting that *Mrgprb2*^high^ MCs represent a mature population of MCs as compared with *Mrgprb2*^low^ population (Fig. S3 E). Altogether, these data strongly suggest that *Mrgprb2*^low^ and *Mrgprb2*^high^ MCs belong to the same population of skin CTMCs but at a different maturation state.

### Mouse CTMCs and MMCs differ in hematopoietic origins and turnover kinetics

**Figure 3.**
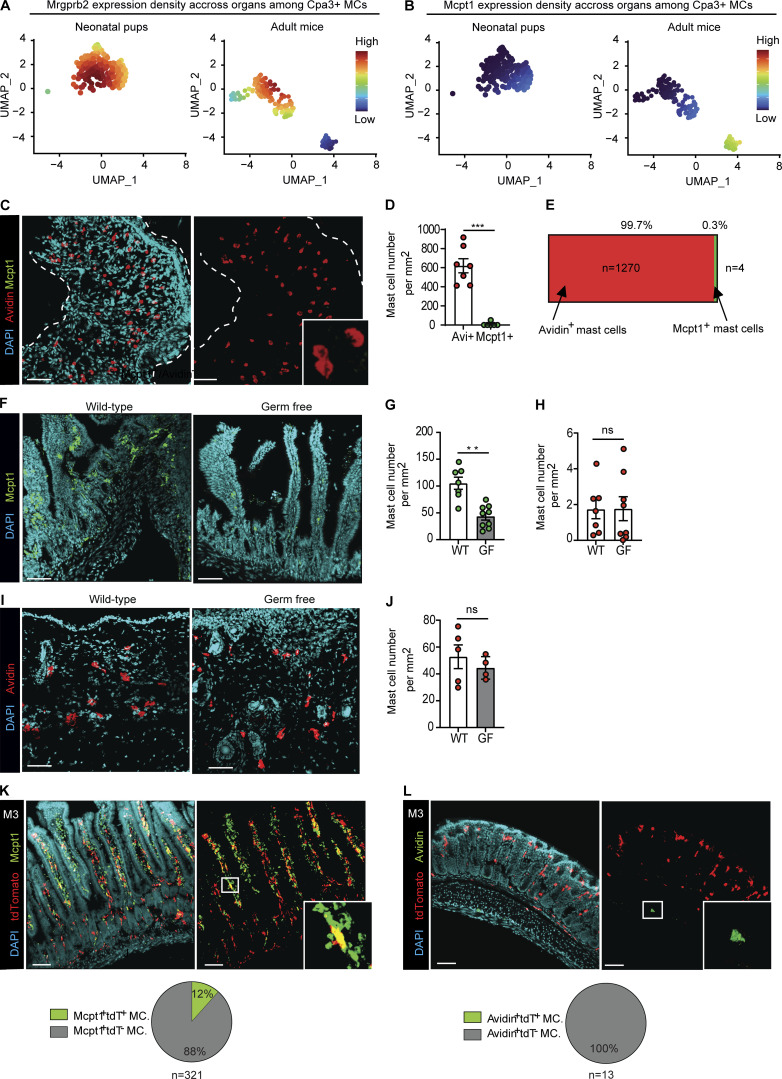
**MrgprB2**^**+**^ **and MrgprB2**^**neg**^ **MCs have different hematopoietic origins and turnover kinetics. (A and B)** *Mrgprb2* (A) and *Mcpt1* (B) expression density in MCs identified in the aggregated scRNAseq data of neonates (left) or adult (right) mice. **(C)** Representative 3D confocal microscopy images of Avidin SRho (red), Mcpt1 (green), and DAPI (cyan) fluorescent signals of E18 neonatal skin. **(D)** Avidin^+^ and Mcpt1^+^ MCs counts in skin samples from seven E18 embryos. **(E)** Bar graph representing the ratio of Mcpt1^+^/Avidin^+^ MCs among gut segments. **(F)** Representative 3D confocal microscopy images of Mcpt1 (green) and DAPI (cyan) fluorescent signals in small intestine of conventionally housed (left) or germ-free (GF, right) mice. **(G and H)** Mcpt1^+^ MCs count in the mucosa (G) and Av.SRho^+^ MCs in the muscularis (H) of conventionally housed (*n* = 7) or GF (*n* = 9) mice. **(I)** Representative 3D confocal microscopy images of Avidin (green) and DAPI (cyan) fluorescent signals in skin of conventionally housed (left) or GF (right) mice. **(J)** Avidin^+^ MCs count in the mucosa of conventionally housed (*n* = 5) or GF mice (*n* = 4). **(K)** Representative 3D confocal microscopy images of Mcpt1^+^ MCs (green), Tdt (red), and DAPI (cyan) in the mouse GI tract, 3 mo (M3) after BM transfer. Pie chart representation of the partition of Mcpt1^+^ Tdt^+^ MCs (green) or Mcpt1^+^ Tdt^−^ MCs (gray) 3 mo after transplantation. **(L)** Representative 3D confocal microscopy images of Avidin^+^ (green), Tdt (red), and DAPI (cyan) in the mouse GI tract, 3 mo (M3) after BM transfer. Pie chart representation of the partition of Avidin^+^ Tdt^+^ MCs (green) or Avidin^+^ Tdt^−^ MCs (gray) 3 mo after transplantation. Scale bars = 50 µm (C–I) and 80 µm (K and L). Data from at least two independent experiments, with at least three mice per experiment mean ± SEM; **P < 0.01; ***P < 0.001, Mann–Whitney test.

Previous fate mapping studies revealed a conserved dual hematopoietic origin of MCs, notably with skin MCs deriving from both hematopoietic progenitors from the yolk sac and HSCs produced in the aorta-gonado-mesonephros (i.e., adult-type definitive HSCs that ultimately settle in the BM; Gentek et al., 2018; Li et al., 2018). We reanalyzed the previously generated multiorgan dataset of MC populations (Fig. 2) based on the fact that the cells were isolated from pups at birth (neonatal) or adult animals. While we found that *MrgprB2*^*+*^ CTMCs were present in both neonatal pups and adults (Fig. 3 A), *MrgprB2*^*neg*^ (*Mcpt1*^*+*^) MMCs were only detected in adult mice (Fig. 3 B). Importantly, we did not detect the presence of MCs in the neonatal gut datasets. These data strongly suggest that MrgprB2^+^ CTMCs are of embryonic origin while MrgprB2^neg^ MMCs apparently develop after birth. In line with this hypothesis, we found that the skin of embryonic day 18 (E18) embryos was mostly composed of Avidin^+^ Mcpt1^neg^ CTMCs (Fig. 3, C–E). These data are in line with two earlier studies identifying MCs in fetal/neonatal tissues based on fluorescent avidin by flow cytometry (Gentek et al., 2018; Li et al., 2018).

Previous observations have suggested that germ-free mice could exhibit reduced numbers of intestinal MCs (Schwarzer et al., 2019). Because MrgprB2^neg^ MMCs, but not MrgprB2^+^ CTMCs, seemed to preferentially develop after birth and are often located in the gut lamina propria in close proximity to the colonizing microbiome, we hypothesized that the microbiota could regulate MrgprB2^neg^ MC development and we therefore searched for both MC populations in WT versus germ-free adult mice. We observed significantly reduced numbers of Mcpt1^+^ MC numbers in the GI tract of germ-free mice compared with WT mice (Fig. 3, F and G), whereas the numbers of Avidin^+^ CTMCs in the gut muscularis (Fig. 3 H) or the skin (Fig. 3, I and J) were essentially unchanged. These results demonstrate that MrgprB2^neg^ MMCs, but not MrgprB2^+^ CTMCs are, at least in part, dependent on microbiome-derived signals for full development.

**Figure S4.**
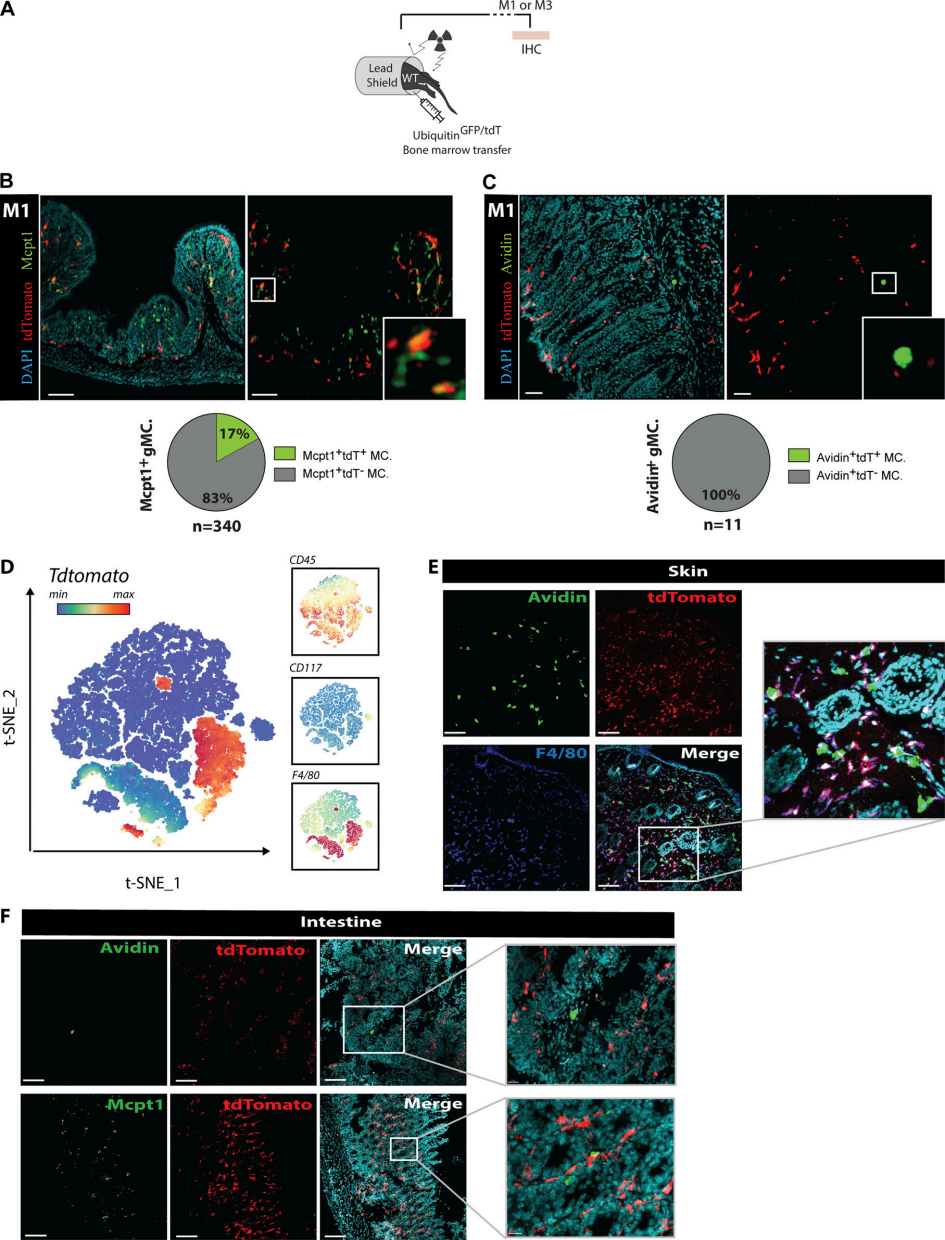
**Ontogeny and renewal of Mrgprb2**^**+**^ **and Mrgprb2**^**−**^ **MCs. (A)** Protocol of the shielded BM chimera strategy used to track cell renewal. **(B and C)** Representative 3D confocal microscopy images (upper panel) and pie chart representation of the partition (lower panel) of (B) Mcpt1^+^ gMCs (green, left panel) and (C) Avidin^+^ gMCs (green, right panel), tdT (red), and DAPI (cyan) in the mouse GI tract, 1 mo (M1) after BM transfer. White squares identify magnified areas shown in the right images (B and C). Number of mice: B and C, *n* = 6, data from two independent experiments. **(D)** t-SNE of the Tdt expression in CD45^+^ immune cells from peritoneal lavage in *Ms4a3-cre*^*+*^*; Tdtomato* mice. Insets on the right show CD45 (upper panel), CD117 (middle panel), and F4/80 (lower panel) expression. **(E)** Representative 3D confocal microscopy images of Avidin^+^ MCs (green), tdT (red); F4/80 (blue) in the skin of *Ms4a3-cre*^*+*^*; tdTomato* mice. **(F)** Representative 3D confocal microscopy images of Avidin^+^ gMCs (green, upper panel) and Mcpt1^+^ gMCs (green, lower panel), tdTomato (red), and DAPI (cyan; upper panel) in the intestinal tract of *Ms4a3-cre*^*+*^*; tdTomato* mice. White squares identify magnified areas shown in the right images. Scale bars = 100 µm. Number of mice: D–F, *n* = 4, data from two experiments.

MCs in the skin and peritoneal cavity (i.e., identified as MrgprB2^+^ MCs in our datasets) have been shown to be independent of BM-derived cells for renewal (Gentek et al., 2018; Li et al., 2018). We next used shielded BM chimeras (Baratin et al., 2017; Scott et al., 2016) that capture homeostatic immune cell turnover to investigate the homeostasis of MrgprB2^+^ versus MrgprB2^neg^ MCs within the same organ, the gut, to avoid any tissue-dependent bias in cell repopulation (Fig. S4 A). We found that Av.SRho^neg^ Mcpt1^+^ lamina propria (i.e., MrgprB2^neg^) MMCs contained a significant fraction of donor-derived cells at 1 and 3 mo after BM transplantation (Fig. 3 K and Fig. S4 B). Remarkably, we did not detect any donor-derived cells among the Av.SRho^+^ Mcpt1^neg^ (i.e., MrgprB2^+^) CTMCs after BM transplantation (Fig. 3 L and Fig. S4 C). This confirmed that the MrgprB2^+^ CTMC population is independent of BM-derived cells for renewal not only in the skin and peritoneal cavity but also in the gut.

Flow cytometry–based analyses have described common MC/basophil BM-derived progenitors among granulocyte–monocyte progenitors (GMPs) that are thought to give rise to MCs in various tissues (Dahlin and Hallgren, 2015; Gurish and Boyce, 2006). We, therefore, used 8–10-mo-old GMP fate-mapper *Ms4a3-Cre; Rosa*^*Tdt*^ mice to specifically trace whether MrgprB2^neg^ or MrgprB2^+^ MCs could originate from and/or be renewed by Ms4a3^+^ GMPs (accounting for 80% of all GMPs; Liu et al., 2019). In line with previous findings (Liu et al., 2019), GMP-derived monocytes and a significant population of monocyte-derived macrophages were positive for Tdt. In contrast, neither MrgprB2^+^ nor MrgprB2^neg^ MCs (even though this last population depends on BM-derived cells for development/turnover) were found to express Tdt (Fig. S4, D–F).

Taken together, these results indicate that MrgprB2^neg^ and MrgprB2^+^ MC subsets have distinct developmental origins and turnover kinetics. MrgprB2^+^ CTMCs appear to develop during embryogenesis and are independent of BM-derived progenitors for turnover. Conversely, MrgprB2^neg^ MMCs develop after birth, are partially dependent on microbiome-derived signals, and are continuously renewed (at least on a monthly basis) from Ms4a3^neg^ non-GMP BM-derived progenitors.

### Partial depletion of the MrgprB2^+^ CTMCs protects against anaphylactic shock induced by food allergens

**Figure 4.**
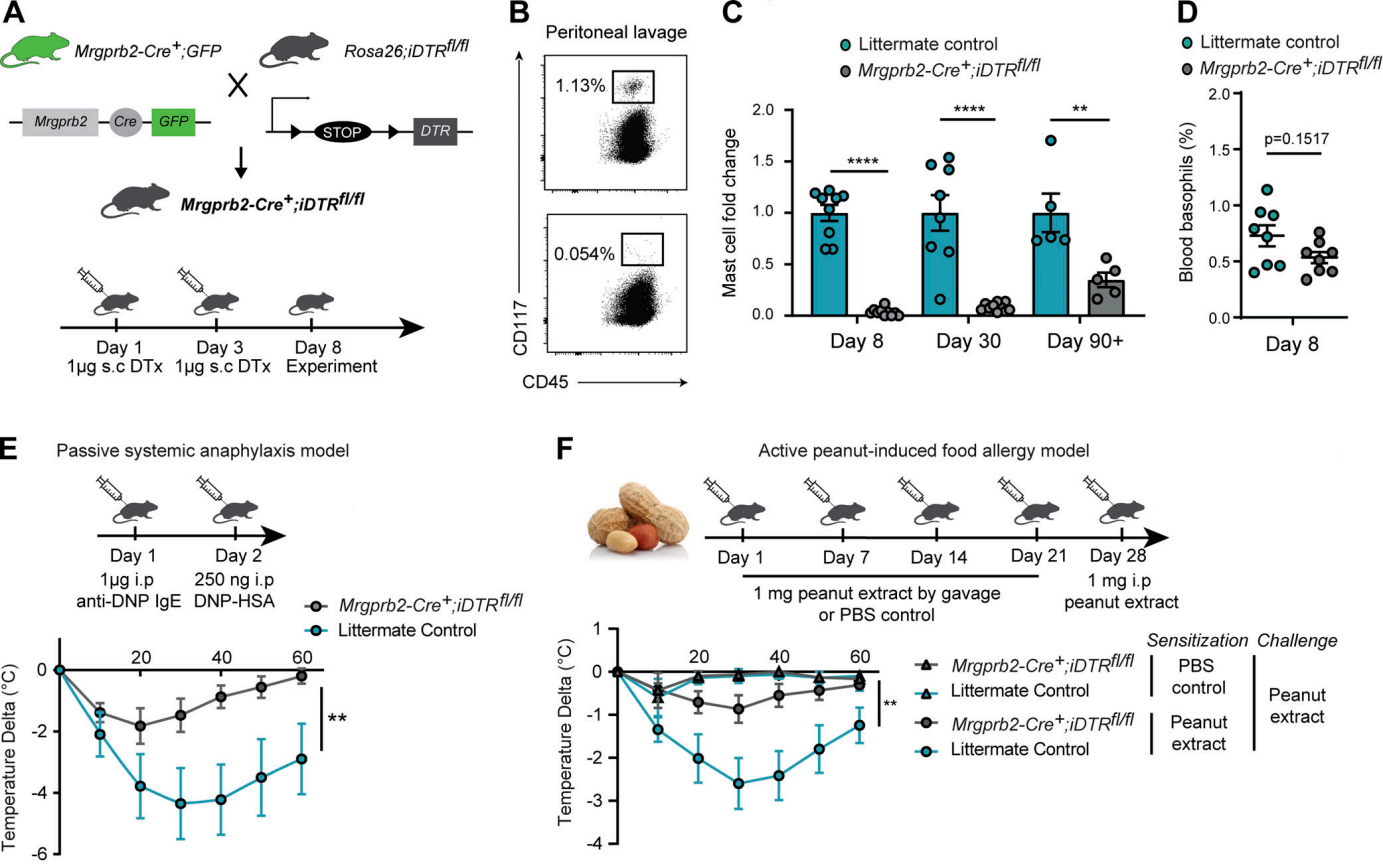
**Selective depletion of MrgprB2**^**+**^ **MCs protects against passive and active anaphylaxis. (A)** Protocol used to selectively deplete MrgprB2^+^ MCs in 6–8-wk-old mice. Two consecutive i.p. injections of 1 µg dt (DTx) were done on days 1 and 3 *Mrgprb2-Cre*^*+*^*; iDTR*^*fl/fl*^ mice versus littermate controls. **(B)** Detection of MCs (CD45^+^ CD117^+^) by flow cytometry in the peritoneal cavity after dt treatment. **(C)** Fold change in MC percentage in the peritoneal lavage at 8, 30, and 90–120 d after dt treatment. **(D)** Percentage of blood basophils 8 d after dt treatment. **(E)** Protocol used to induce passive systemic anaphylaxis in *MrgprB2-Cre; iDTR*^*fl/fl*^ (gray circles, *n* = 8) or littermate controls (blue circles, *n* = 8) mice. Mice were treated i.p with 1 µg of anti-DNP IgE 24 h followed by i.p injection of 250 ng of DNP-HSA. Anaphylactic response was monitored by assessment of rectal temperature every 10 min for 60 min. Results are expressed as change in temperature over time. **(F)** Protocol used to induce peanut-induced food allergy anaphylaxis in *MrgprB2-Cre; iDTR*^*fl/fl*^ (gray circles, *n* = 12) or littermate control (blue circles, *n* = 8) mice. Mice were sensitized to peanut by weekly gavage for 4 wk with 1 mg of peanut extract and cholera toxin. 1 wk after the last gavage, mice were challenged i.p. with 1 mg of peanut extract. Anaphylactic response was followed by assessment of rectal temperature every 10 min for 60 min. Results are expressed as change in temperature over time. Non-sensitized *MrgprB2-Cre; iDTR*^*fl/fl*^ (gray triangles, *n* = 5) or littermate controls (blue circles, *n* = 3) were used as controls. Data are from at least two independent experiments, with at least two mice per group; mean ± SEM; **P < 0.01, ****P < 0.0001 two-way ANOVA.

**Figure S5.**
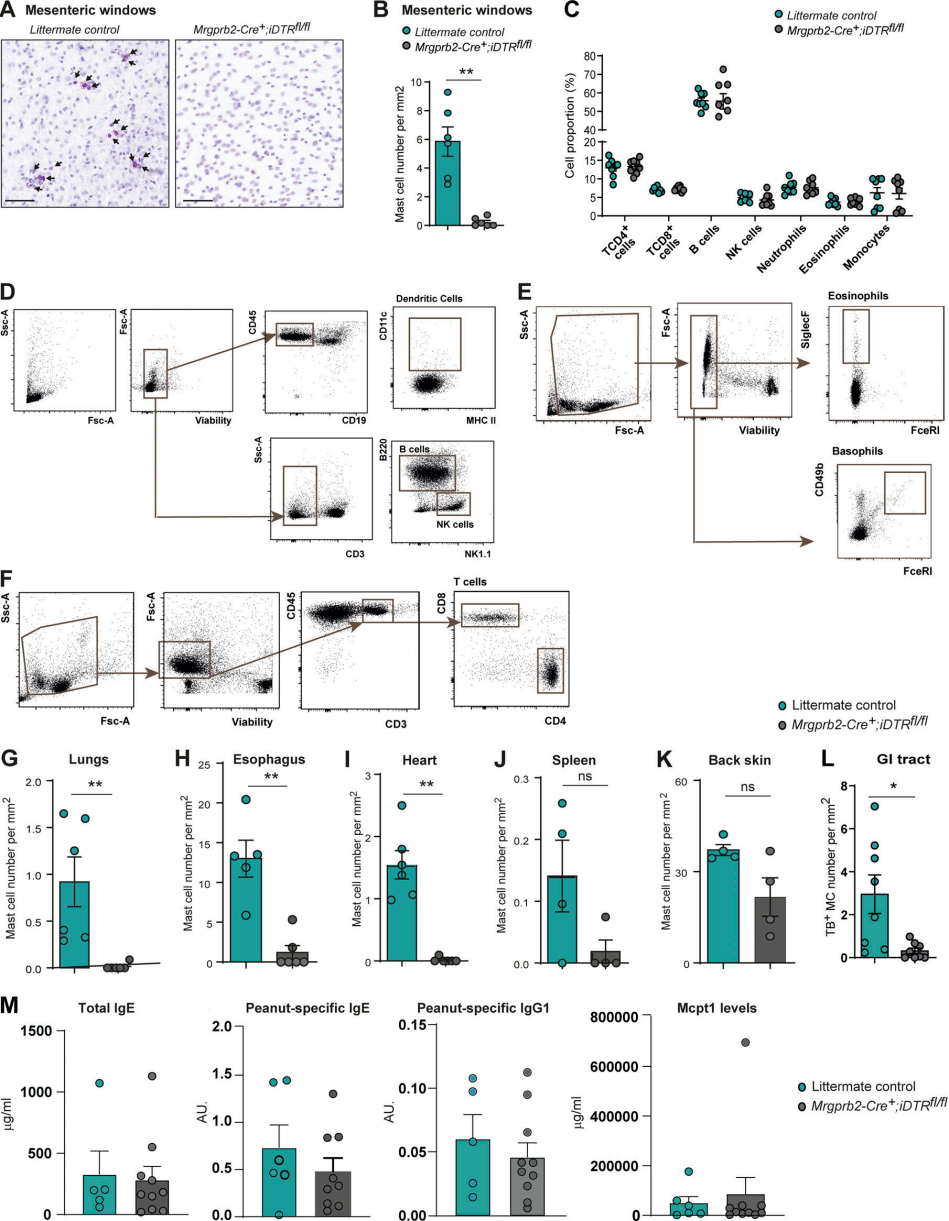
**Selective depletion of MrgprB2**^**+**^ **MCs achieved across organs does not impact other immune cells. (A)** Representative TB staining photographs of mesenteric windows (MW) from *Mrgprb2-Cre*^*+*^*; iDTR*^*fl/fl*^ mice (right panel) and littermate controls (left panel) after two i.p. dt injections. Black arrows indicate MCs. Scale bars = 100 µm. **(B)** MCs count in the MW of *Mrgprb2-Cre*^*+*^*; iDTR*^*fl/fl*^ mice (gray bar) and the littermate controls (cyan bar) based on TB staining. **(C)** Percentage of blood lymphocytes (CD4^+^ T cells, CD8^+^ T cells, B cells), natural killer (NK) cells, neutrophils, eosinophils, monocytes in *Mrgprb2-Cre*^*+*^*; iDTR*^*fl/fl*^ mice (gray circles), and the littermate controls (blue circles) after dt treatment (day 8), gating by flow cytometry. **(D–F)** Gating strategy for immune cell populations analyzed by flow cytometry in C. **(G–K)** MC count in the lungs (G), esophagus (H), heart (I), spleen (J), back skin (K), and GI tract (L, pooled jejunum, ileum, and colon sections) of *Mrgprb2-Cre*^*+*^*; iDTR*^*fl/fl*^ mice (gray bars) and the littermate controls (cyan bars) after two i.p. dt injections, based on TB staining. **(M)** Total IgE, peanut specific IgE, peanut specific IgG1, and Mcpt1 levels in blood of littermates (cyan bars, *n* = 5–6) or *Mrgprb2-Cre*^*+*^*; iDTR*^*fl/fl*^ mice (gray bars, *n* = 10) sensitized and challenged with peanut extract. **(B, C, and G–M)** Each circle = one mouse. Data from at least two independent experiments, mean ± SEM; *P < 0.05, **P < 0.01 Mann–Whitney test.

We next aimed to establish an inducible method to selectively deplete MrgprB2^+^ CTMCs in adult mice. We generated *Mrgprb2-Cre*^*+*^*; iDTR*^*fl/fl*^ transgenic mice in which the gene encoding the diphtheria toxin (dt) receptor is placed under the control of the *Mrgprb2* promotor to selectively deplete MrgprB2^+^ cells upon injection of dt in vivo. We established a protocol based on two consecutive injections of 1 μg of dt on days 1 and 3 in 8-wk-old *Mrgprb2-Cre*^*+*^*; iDTR*^*fl/fl*^ mice versus littermate controls and analyzed the presence of MCs in various organs on day 8 (Fig. 4 A). We found that most peritoneal MCs (including those located in mesenteric windows) were efficiently depleted upon injection of dt (Fig. 4, B and C; and Fig. S5, A and B) and then were slowly replenished with only 50% of MCs restored within 90–120 d after dt injection (Fig. 4 C). Importantly, such MC depletion did not significantly affect the percentage of blood immune cell populations, including basophils (Fig. 4 D and Fig. S5, C–F). The dt treatment also depleted most toluidine blue (TB)^+^ MCs in the lungs, esophagus, heart, and spleen, and reduced MC numbers in the skin (Fig. S5 G–L), confirming the presence of MrgprB2^+^ CTMC in these organs.

Food-induced systemic anaphylaxis is a potentially life-threatening form of allergy for which pathological features are often observed in many tissues such as the GI tract, skin, lungs, and cardiovascular system (Dahlin et al., 2022; Reber et al., 2017a, 2017b). Allergen-specific IgE antibodies and MCs are thought to contribute importantly to the development of the pathological features of systemic anaphylaxis. When triggered by orally ingested food allergens, the reaction is usually initiated within the buccal cavity, then spread all along the GI tract and subsequently to the whole organism within a few minutes after ingestion by the patient. Previous studies in mice have shown that allergy models, including food allergy, triggered a significant expansion of intestinal MMCs, suggesting their role in the development of pathological features associated with anaphylaxis (Burton et al., 2013; Leyva-Castillo et al., 2019; Nakano and Kitaura, 2022). We investigated the role of MrgprB2^+^ versus MrgprB2^neg^ MC populations by assessing *Mrgprb2-Cre*^*+*^*; iDTR*^*fl/fl*^ mice versus littermate controls in a model of IgE-dependent passive systemic anaphylaxis (Fig. 4E) and in a more physiological model of active peanut-induced food allergy over a period of 28 d (Fig. 4 F). In both models, we found that MrgprB2^+^ CTMC-depleted mice were almost completely protected from the development of anaphylactic shock, which in MrgprB2^+^ CTMC-sufficient mice is characterized by a striking drop in body temperature upon challenge with the offending allergen (Fig. 4, E and F). Importantly, the depletion of MrgprB2^+^ CTMC did not affect the circulating levels of peanut-specific IgE and IgG1, total IgE, and the MrgprB2^neg^ MMC protease Mcpt1 (Fig. S5 M). These results show that the partial depletion of Mrgprb2^+^ CTMC was sufficient to largely protect the mice in two models of anaphylactic shock and therefore demonstrate that the MrgprB2^neg^ MMC population found in the gut, albeit being one of the first in contact with food allergens during antigen/allergen challenge, is largely dispensable for the rapid drop of temperature typical of anaphylaxis in these two models.

### Integrated single-cell analysis of human organs identifies six distinct MC states

**Figure 5.**
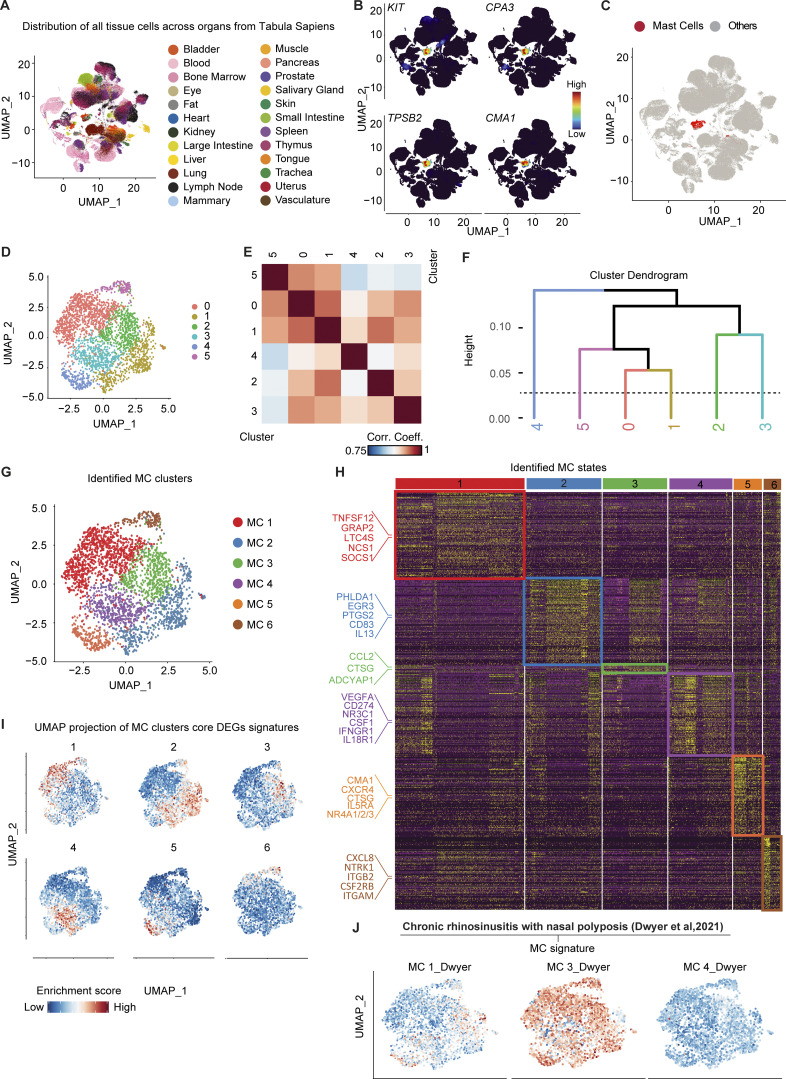
**Transcriptomic profiling of human MCs across tissues. (A)** UMAP plot of the distribution of all tissue cells across organs from the Tabula Sapiens. **(B)** UMAP plot of the expression density of *CPA3*, *KIT*, *TPSB2*, and *CMA1* on the cells from the Tabula Sapiens. **(C)** UMAP showing the final MC clusters selected for further clustering. **(D)** UMAP of the Louvain clustering of the selected MCs. **(E)** Heatmap of correlation between the six Louvain communities after pseudo-bulk transformation. **(F)** Hierarchical clustering of Louvain communities based on the distance of correlation. Populations were grouped if the distance between them was inferior to 0.1 (dotted line). **(G)** UMAP showing the final six states of human MCs identified. **(H)** Heatmap of 350 representative DEGs between the six populations of MCs. Genes of interest of each population are highlighted on the left. **(I)** UMAP showing the score-based identification of each of the six states using a selected set of markers. **(J)** UMAP showing the score-based identification of the six states of human MCs using the signature of MC1, MC2, and MC4 populations defined in Dwyer et al. (2021).

Of relevance for drug development and MC-related therapies, we next investigated the transcriptomic heterogeneity of human MC populations across different organs. We aggregated the databases of 24 different organs from the Tabula Sapiens, which is already decontaminated from ambient RNA with Decontx (Yang et al., 2020), as part of the processing guidelines (https://tabula-sapiens-portal.ds.czbiohub.org/), in a unique UMAP composed of 264,009 single cells (Fig. 5 A). Taking into account differences in gender and sequencing technologies, we applied two additional layers of integration (detailed in the Materials and methods section) to avoid any potential bias of analysis. We next identified a unique cluster composed of 2,690 MCs based on the signature of the cardinal MC genes *KIT*, *CPA3*, *TPSB2*, and *CMA1* (Fig. 5, B and C)*.* We then projected all of the identified MCs on the same UMAP, reaching a total number of 2,690 single MCs from 12 different organs. The human datasets appear to be more complex and heterogeneous than the mouse datasets, and we could not observe an obvious CTMC/MMC transcriptomic dichotomy in humans based on the expression of the classical histochemical markers *CMA1* and *TPSB2* reported in the literature (Derakhshan et al., 2022).

We, therefore, decided to adopt an unbiased approach to better understand human MC heterogeneity and performed an unsupervised nearest-neighbor analysis that identified the presence of six potential clusters (Fig. 5 D). To decipher the number of MC subsets present among these six clusters, we generated a correlation heatmap (Fig. 5 E) followed by a cluster dendrogram (Fig. 5 F) to directly visualize the strength of relationships between the different clusters. We could confirm the presence of six potential MCs clusters (Fig. 5 G) with a total of 1,570 statistically significant DEGs that defined the transcriptomic heterogeneity between each cluster of MCs (Fig. 5 H). The total list of DEGs characteristic to each of the human MC clusters, hereafter named MC1–6, is provided in Table S3.

Among the many DEGs, MC1 notably expressed genes encoding the TNF superfamily member 12 (*TNFSF12*), the leukotriene C4 synthase (*LTC4S*), the suppressor of cytokine signaling 1 (*SOCS1*), and the GRB2-related adaptor protein 2 (*GRAP2*). MC2 notably were characterized by the expression of genes encoding interleukin 13 (*IL13*), the pleckstrin homology-like domain family A member 1 (*PHLDA1*), the early growth response 3 gene (*EGR3*), prostaglandin-endoperoxide synthase 2 (*PTGS2*), and the cluster differentiation 83 (*CD83*). MC3 expressed, among others, the cathepsin G (*CTSG*), the gene encoding the neuropeptide PACAP (*ADCYAP1*), and the chemokine CCL2. MC4 expressed genes encoding growth factors (*VEGFA, CSF1*), the glucocorticoid receptor (*NR3C1*), and cytokine receptors (*IFNGR1*, *IL18R1*). MC5 was preferentially enriched in genes encoding the chymase 1 (*CMA1*), the chemokine receptor *CXCR4*, the cathepsin G (*CTSG*), IL5 receptor alpha (*IL5RA*), and multiple members of the nuclear receptor subfamily 4 group A (*NR4A1/2/3*). Finally, MC6 was preferentially enriched in genes encoding integrins (*IGAM, ITGB2*), neuronal growth factor receptor (*NTRK1*), and the common unit of IL3 and IL5 receptors (*CSF2RB*). Interestingly, *MRGPRX2* was found heterogeneously expressed among clusters with a tendency for enrichment in MC5 and MC2 but, *MRGPRX1*, another receptor reported to be the human ortholog of *Mrgprb2*, could not be found in any dataset.

We then isolated each MC’s DEGs signature (Table S4) and isolated a reduced list of 10 genes to establish a “core signature” that would identify each MC state. We then projected the score of each of these “core signatures” on the aggregated UMAP of MCs (Fig. 5 I). Using this approach, we could confirm that each identified set of DEGs enabled the precise identification of the corresponding MCs in the UMAP.

Finally, we extracted scRNAseq transcriptomic signatures from the states of MCs identified in chronic rhinosinusitis with nasal polyposis patients by Dwyer et al. (2021) and projected them on our aggregated UMAP of MCs (Fig. 5 J). We could show that the MC_3 populations identified by Dwyer et al. (2021) matched several of our populations whereas their MC1 signature matched two discrete MC clusters in our datasets, namely MC2 and MC5 clusters. These data demonstrate that at least six MC clusters/states with distinct transcriptomic signatures exist across organs in humans and strongly suggest that the heterogeneity of human MCs might extend far beyond the classical CTMC/MMC dichotomy.

### Distribution of the six MC states across organs in human datasets

**Figure 6.**
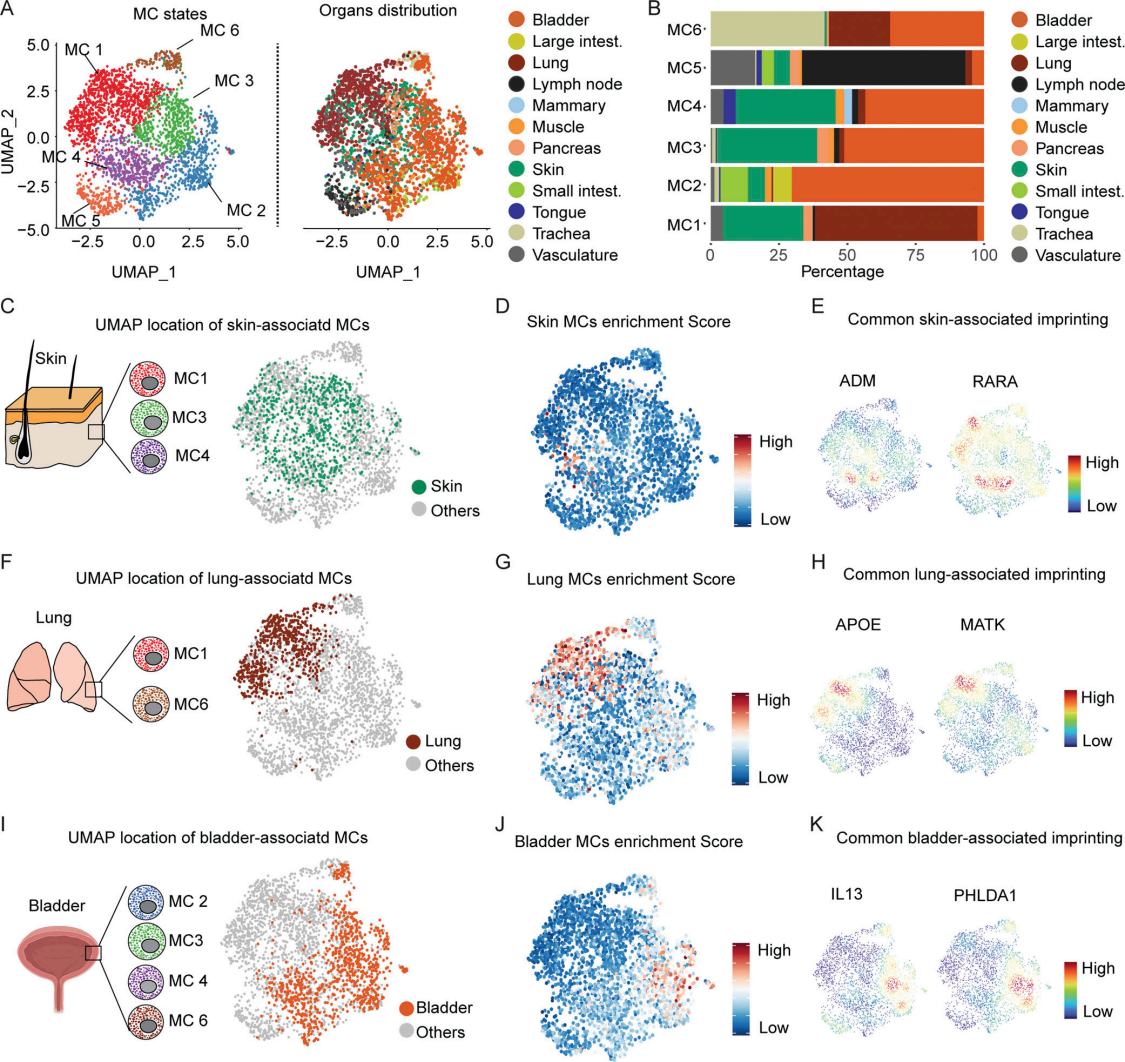
**Distribution of human MC states across organs and genes expression in major subsets from the same organ. (A)** UMAP representing the human MCs colored according to the six identified clusters (left UMAP) or the organ of origin (right UMAP). **(B)** Bar graph showing the proportion of each MC states in the different organs. **(C)** Picture showing the MC cluster present in the skin (left) and their distribution on the UMAP (right). **(D)** UMAP showing the score-based identification of skin MCs using a common set of markers from MC1, MC3, and MC4 states. **(E)** Examples of UMAPs showing the expression density of common sets of skin-associated genes. **(F)** Picture showing the MCs present in the lung (left) and their distribution on the UMAP (right). **(G)** UMAP showing the score-based identification of lung MCs using a common set of markers from MC1 and MC6 populations. **(H)** Examples of UMAPs showing the expression density of lung-associated genes. **(I)** Picture showing MC states present in the bladder (left) and their distribution on the UMAP (right). **(J)** UMAP showing the score-based identification of bladder MCs using a common set of markers from MC2, MC3, MC4, and MC6 states. **(K)** Examples of UMAPs showing the expression density of bladder-associated genes.

We then investigated the anatomical distribution of the six MC clusters in the different human organs (Fig. 6 A). We found that the six MC clusters were heterogeneously distributed among different organs, but some organs displayed restricted representations of MC states, such as the large and small intestines (MC2 and MC5 only), lymph node (majority of MC5), and lung (MC1 and MC6) (Fig. 6 B).

The distribution pattern of the different MC states prompted us to investigate potential common makers among them. We first investigated whether different MC subsets could express common gene expression features when located in the same tissue. We analyzed the common gene expression pattern of MC1, MC3, and MC4 to identify DEGs previously reported to be important players in skin homeostasis (Fig. 6 C). We created an enrichment score (the list of genes is provided in Table S5) that could reflect a potential skin-related biological process and projected it on the aggregated UMAP of all MCs population. We found that such a genes signature was restricted to those MCs found in the skin but not in other organs (Fig. 6 D), among which were *ADM*, *RARA*, and *LAMA5*, which encode key components of skin homeostasis (Meixiong et al., 2019; Szymański et al., 2020; Wegner et al., 2016; Fig. 6 E).

Because the lungs were found to be composed of MC1 and MC6 clusters, we, therefore, investigated the presence of DEGs previously reported to be involved in lung homeostasis (Fig. 6 F). We created a lung MC enrichment score (Table S5) and projected it on the aggregated UMAP representing all MC populations. We found a common signature of genes among the three MC subsets that was restricted to those MCs found exclusively in the lungs (Fig. 6 G), among which were *APOE* and *MATK* (megakaryocyte-associated tyrosine kinase), both previously reported to play a role in lung diseases (Gordon et al., 2019; Xiao et al., 2012; Zhong et al., 2021; Fig. 6 H). We performed the same experiment with the bladder, which is composed of MC2, MC3, MC4, and MC6 (Fig. 6 I and Table S5). In such MC states, we notably found a common signature of genes restricted to the bladder (Fig. 6 J), among which are *IL13* and *PHLDA1* (Fig. 6 K). These data strongly suggest that different MC states found in the same organ could share the expression of common genes involved in the homeostasis of the tissue in which they reside.

## Discussion

### Heterogeneity and distribution of MCs in different anatomical locations in mice and humans

Previous histochemical studies have classified MCs into two categories, CTMCs and MMCs. CTMCs referred to MCs found largely in the skin (Befus et al., 1985; Katz et al., 1985), while MMCs were reported to populate the mucosa of the gut and have lower amounts of histamine (Befus et al., 1985; Enerbäck, 1966a, 1966b; Katz et al., 1985). These two MC populations also differed in their repertoire of granule-associated proteases (Gurish and Austen, 2012; Stevens and Austen, 1989). However, a current challenge in the field of MC biology is to understand the ontogeny and full heterogeneity of MC subsets across tissues. In this study, we confirmed and extended the previous CTMC and MMC classification by exploring the phenotypical and functional heterogeneity of MCs both in mice and humans. Using in silico investigation, we projected single-cell profiles of MCs from different organs in mice and in humans. To avoid bias inherent to both scRNAseq technic and the merging of multiple scRNAseq datasets from multiple origins, we performed removal of contamination from ambient RNA using the DecontX package (Yang et al., 2020) on each individual dataset and integrated all the data using the Harmony package (Korsunsky et al., 2019) as per recommendations of the current best practice for analysis of the scRNAseq experiments (Luecken and Theis, 2019).

In mouse, we found two major MC clusters with almost 200 DEGs, confirming that previously described CTMCs and MMCs represent two clearly distinct MC populations. While we observed that MrgprB2^+^ CTMCs were present in virtually all analyzed organs, MrgprB2^neg^ MMCs were exclusively found in the lamina propria of the GI tract. As previously suggested by the presence of MrgprB2^+^ MCs in the skin (McNeil et al., 2015), it thus appears that MrgprB2 represents the first surface marker to specifically distinguish “CTMC-like” and “MMC-like” MC populations in mouse single-cell datasets. Notably, a previous study has reported the presence of integrin β7^high^ and β7^low^ MCs in the lungs of mice during the development of a model of allergic airway inflammation (Derakhshan et al., 2021). Based on bulk RNAseq data on sorted MCs, it is likely that β7^high^ and β7^low^ lung MCs might be related to MrgprB2^neg^ MCs and MrgprB2^+^ MCs, respectively.

Gut MMCs remain incompletely understood due, at least in part, to the lack of genetic approaches to specifically target them and therefore are often ignored by immunologists working on other myeloid cells. Based on our characterization of the gut MMC transcriptome, among the most significant DEGs compared with CTMCs is *Cx3cr1*, a classical marker associated with macrophages. Many studies have used *Cx3cr1-Cre* mice to specifically deplete or label macrophages and understand their function in vivo. The extent to which these mice allow a simultaneous labeling/depletion of gut (and potentially lung) MMCs is currently unknown.

We used the large publicly available dataset from the Tabula Sapiens consortium to generate an aggregated UMAP composed of cells from 24 human organs. From this UMAP, we used the canonical MC markers *KIT*, *CPA3*, *TPSB2*, and *CMA1* to extract the cluster of MCs. When we analyzed the expression profile of *TPSB2* and *CMA1*, we could not find a clear CTMC (i.e., positive for *TPSB2* and *CMA1* or *MRGPRX2*) versus MMC (i.e., positive only for *TPSB2*; at least according to previous literature based on histochemical analyses). We, therefore, decided to take an unbiased approach and identified the presence of six distinct MCs clusters based on the presence of many DEGs between these clusters (with MC2 and MC3 being relatively close to each other). Importantly, we mentioned the word “cluster” or “state” to qualify the distinct MC transcriptomic profiles identified, as it is still unclear whether they represent different transcriptomic states of MCs or truly different MC subsets with different origins and/or renewal dynamics.

It thus appears that the complexity and transcriptomic heterogeneity of human MCs goes beyond the classical CTMC/MMC dichotomy that we observed in mice, and that, among what have been called CTMCs or MMCs in humans, there should be different varieties of MCs with distinct transcriptomic programs. Among the six different human MC clusters/states that we identified in this study, they do not seem to be restricted to a single organ. Our classification also adds a layer of complexity to the previous study that identified four different populations of MCs by scRNAseq in polyps from patients suffering from chronic rhinosinusitis (Dwyer et al., 2021).

A very important question that will need to be addressed is whether such transcriptomic diversity in human MCs also reflects a broad spectrum of specialized biological functions. Interestingly, whether mouse and human MCs could fulfill potential microenvironment-specific functions such as tissue defense or support remains to be investigated.

### Differential origin and renewal dynamics of MCs

Skin MCs have been reported to be of dual origin in the embryo (Gentek et al., 2018; Li et al., 2018). Here, our study revealed a higher level of complexity among MC populations. We confirmed that Avidin^+^ (MrgprB2^+^) CTMCs indeed might develop during embryogenesis and are independent of BM-derived progenitors for renewal. It is thus interesting to speculate that MrgprB2^+^ CTMCs could be considered “self-renewing,” like certain populations of gut macrophages (De Schepper et al., 2018; Gabanyi et al., 2016), and/or “long-lived” (at least more than 3 mo). Conversely, we demonstrated that MrgprB2^neg^ MCs are only detectable after birth, are largely dependent on microbiome-derived signals for full development, and are partially renewed after 1 mo by BM-derived progenitors (i.e., being relatively “short-lived” compared to their MrgprB2^+^ counterpart). Clearly, the identification of which microbiome species are required for the development of gut-associated MMCs will be an intriguing area of exploration.

Previous studies have reported that a common basophil/MC progenitor arising from GMPs could give rise to MCs, at least in the mouse (Dahlin and Hallgren, 2015; Gurish and Boyce, 2006). Using a fate mapping model based on the transient expression of *Ms4a3* in GMPs, we here found that neither MrgprB2^+^ CTMCs nor MrgprB2^neg^ MMCs (albeit MrgprB2^neg^ MMCs deriving from BM progenitors) are dependent on Ms4a3^+^ GMPs for development/renewal at steady state. These results strongly suggest that the MrgprB2^neg^ MMC population develops after birth from Ms4a3^neg^ GMPs (representing 20% of all GMPs; Liu et al., 2019) or from a progenitor that emerges from the common myeloid progenitor and diverges before the GMP state. Further investigations are needed to better understand the precise ontogeny of the MrgprB2^neg^ MMCs. Nevertheless, our data highlight the key roles played by the origin, tissue of residence, and microbiome-derived factors in imprinting the transcriptomic profile of MCs and determining their subsequent function.

In the gut, MrgprB2^+^ CTMCs are deeply anchored within the muscularis propria while MrgprB2^neg^ MCs are restricted to the lamina propria. Such dichotomies in the capacity of the two MC populations to colonize particular anatomical niches and in their longevity might reflect the specific biological functions of each population. Previous reports have shown that the MC protease Mcpt1 (found exclusively in MrgprB2^neg^ MMCs in our study) plays a protective role during parasite infections by notably reducing intestinal inflammation (Knight et al., 2000; Lawrence et al., 2004). While other studies used *Mcpt-5-cre*; *iDTR*^*fl/fl*^ mice (Reber et al., 2013; Scholten et al., 2008) to target CTMCs, in this study, we performed a detailed characterization of *Mrgprb2-Cre*^*+*^*; iDTR*^*fl/fl*^ mice and used such mice to study the intrinsic role played by some MrgprB2^+^ CTMC populations in models of systemic anaphylaxis without affecting MrgprB2^neg^ MMCs or other tested immune cells. We found that MrgprB2^+^ CTMCs play an active role not only in passive systemic anaphylaxis but also in active food-induced anaphylaxis, suggesting that CTMCs could participate in systemic symptoms of food allergy, as already suggested (Reber et al., 2013), while MMCs could participate in GI symptoms like pain, bloating, or diarrhea that are often associated with food allergy (Li et al., 2000). It would be also interesting to assess if, as observed for *Mcpt-5-cre*; *iDTR*^*fl/fl*^ mice, *Mrgprb2-cre*^*+*^; *iDTR*^*fl/fl*^^+^ could be protected from other anaphylaxis models, such as in response to massive nociceptor activation (Bao et al., 2023), especially with regard to the Mrgprb2^+^ MC/nociceptors interactions described in the literature (Serhan et al., 2019).

In conclusion, our study provides a new perspective on the heterogeneity and tissue-specific specialization of MCs both in the mouse and in humans. Altogether, we reveal an unexpected level of transcriptomic heterogeneity, particularly among the human MC populations. Our data support the existence of at least six distinct transcriptionally defined MC clusters in humans in addition to the two main MC populations (i.e., CTMCs and MMCs) in mice. Approaches such as those we have used in this study should be applied to data derived from even more anatomical locations in both mice and humans, and the results may be helpful in defining additional distinct subtypes of MCs in both species. The extent to which and how this can influence organ-level functions and immune responses remain to be investigated. In aggregate, this study constitutes an online resource that regroups multiple MC single-cell transcriptomic profiles from various mouse and human organs and should help to refine MCs annotation and better understand their specialized functions across organs.

## Materials and methods

### Mice

6–8-wk-old C57BL/6J mice were purchased from Charles River Laboratory; both age-matched male and female mice were used (and no gender-related differences were found). *Mrgprb2-Cre* mice (in which the expression of the Cre recombinase is under the control of the *Mrgprb2* promotor) were provided by X. Dong (McNeil et al., 2015). *Ai32(RCL-ChR2(H134R)/EYFP* (also known as Ai32 mice), Ai9 (*B6.Cg-Gt(ROSA)26Sortm9(CAG-tdTomato)Hze/J,* Tac1^−/−^ (*B6.Cg-Tac1tm1Bbm/J*)) mice, and *B6-iDTR* mice were from the Jackson lab. *Mrgprb2-Cre; EYFP* mice (in which the expression of the EYFP is placed under the control of the *Mrgprb2* promotor) were generated by crossing *Mrgprb2-Cre* mice and Ai32 homozygous mice. *Mrgprb2-Cre; Tdtomato* mice (in which the expression of the Tdt is placed under the control of the *Mrgprb2* promotor) were generated by crossing *Mrgprb2-Cre* mice and Ai9 heterozygous mice. *Mrgprb2-Cre*^*+*^*; iDTR*^*fl/fl*^ mice (in which the expression of the dt receptor [DTR] is placed under the control of the *Mrgprb2* promotor) were generated by crossing heterozygous *Mrgprb2-Cre* mice and heterozygous *iDTR*^*fl/fl*^ mice. Mice were bred and housed in the local animal facilities of Centre Régional d'Exploration Fonctionnelle et de Ressources Expérimentales (Toulouse, France), and littermate control mice were used in all experiments. All animal care and experimentation were conducted in France (Gaudenzio Lab, INSERM, University of Toulouse) in compliance with the guidelines of the European Union (86/609/EEC) and the French Committee of Ethics (87/848) policies and with the specific approval from the local ministry-approved committee on ethics in animal experimentation (Ethics Committee UMS006 CEEA-122, project no 13283 2018031416055447V3). 8–12-wk-old male and female mice were used in all experiments. *Ubiquitin*^*tdT*^ and *Ubiquitin*^*gfp*^ mice were kindly provided by M. Bajénoff. *NDST2*^*−/−*^; serglycin^*−/−*^ and 3xKO (*Mcpt4*^*−/−*^*; Mcpt6*^*−/−*^*; Cpa3*^*−/−*^) mice were as described (Åbrink et al., 2004; Forsberg et al., 1999; Grujic et al., 2013) and were kindly provided by G. Pejler. *Ms4a3-Cre; Rosa*^*Tdt*^ mice were kindly provided by F. Ginhoux. 12-wk-old Sox10-Cre^ERT2^;Rosa26tdT (SER93) mice (kindly provided by R. Lasrado and V. Pachnis, Nervous System Laboratory at the Francis Crick Institute, London, UK) were administered tamoxifen (100 µg/g) twice on two consecutive days and the tissue was collected a week later.

### dt-mediated depletion of MC in *Mrgprb2-Cre; DTR* mice

dt (Sigma-Aldrich) was dissolved in PBS and stored at −20°C. Adult mice were injected intraperitoneally (i.p.) twice with 1 μg dt (2 d apart) as previously described for another model of MC-deficient mice (Dahdah et al., 2014). Littermate mice injected with PBS served as controls.

### Passive systemic anaphylaxis

Mice were sensitized by i.p. injection of 1 µg of mouse dinitrophenylated human serum albumin (DNP-HSA)–specific IgE (D8406; Sigma-Aldrich) in 100 µl PBS, and control mice were mock-injected i.p. with 100 µl of PBS. 16 h later, sensitized or non-sensitized control mice were injected i.p. with 250 ng of DNP-HSA (D8406; Sigma-Aldrich), and rectal temperature was measured every 10 min for a period of 60 min.

### Peanut extract preparation

Peanut extract was prepared from partially defatted peanut flour (Bio Planete). A 10% wt/vol suspension of peanut flour in 0.1 M NaHCO_3_ was brought to pH 9 using NaOH (5 M) and agitated overnight at 4°C. The suspension was centrifuged (3,300 *g*, 60 min) to remove non-dissolved debris, and the supernatant containing the dissolved proteins collected. Under constant agitation, the supernatant was slowly brought to pH5 by addition of HCl (5 M) to precipitate proteins and centrifuged (3,300 *g*, 30 min) to pellet the now insoluble protein fraction. Supernatant was discarded and protein pellets were resuspended in an equal volume of NaHCO_3_ (0.1 M) and brought to pH 8.4 by addition of NaOH (5 M) to resolubilize the proteins. Finally, the protein solution was centrifuged (3,300 *g*, 5 min) and the supernatant, the saturated peanut extract, was transferred to a clean container and stored at −20°C until further use. Protein concentration was determined by Bradford assay.

### Peanut-induced anaphylaxis model

Mice were sensitized with 1 mg of peanut extract (homemade, see above) along with 10 μg of cholera toxin (Sigma-Aldrich) in 100 μl of water (HCO3−) administered by means of oral gavage once a week for 4 wk. 1 wk after the last sensitization with peanut extract, mice were challenged with an i.p. injection of 1 mg of peanut extract (homemade) in 200 μl of PBS. Measurements of rectal temperature were performed immediately before (time 0) and every 10 min for a period of 60 min after peanut challenge.

### BM chimeras

Shield irradiation was performed as described (Baratin et al., 2017; Gentek et al., 2018). Briefly, mice anesthetized with a ketamine/xylazine mixture were placed in a 6-mm-thick lead cylinder that only exposed their hind legs, irradiated (9 Gy), and reconstituted with ∼3 × 10^7^ CD11b depleted BM cells. BMs from *Ubiquitin*^*tdT*^ and *Ubiquitin*^*gfp*^ donors were prepared using standard procedures and underwent negative selection using CD11b magnetic microbeads (Miltyeni). Data were normalized and quantified as described previously (Baratin et al., 2017; Gentek et al., 2018).

### Flow cytometry analysis of immune populations

Cell suspensions from mouse skin (ear and back skin), spleen, and lymph nodes were obtained by mechanical dissociation and/or enzymatic digestion. Skin was sampled from 6/8-wk-old mice. Briefly, skin samples were harvested, finely minced with scissors, and digested in RPMI medium (Invitrogen) containing 0.5 mg/ml DNase I (Sigma-Aldrich) and 0.25 mg/ml Liberase DL (Roche). Digestion was performed for 120 min at 37°C under continuous agitation (1,100 rpm) and samples were regularly pipetted up and down to support mechanical dissociation and digestion. Cell suspensions from mouse spleen and lymph nodes were obtained through mechanical dissociation. Cell suspensions from spleen were subjected to red blood cell lysis with classical ACK lysis buffer (Gibco) prior to staining. Blood samples were drawn from the retro-orbital vein and subjected to red blood cell lysis prior to staining. Peritoneal washes were performed by i.p. injection of 5 ml PBS 0.5 mM EDTA buffer and collection of the resulting peritoneal fluid. Digested tissue samples were filtered and single-cell suspensions were blocked with anti-mouse CD16/32 (S17011E, #156604; BioLegend) for 15 min at 4°C. Surface staining was performed in FACS buffer supplemented for 20–30 min at 4°C with the following antibodies: anti-CD45-APC (30-F11, MCD4505; Invitrogen), anti-CD45-BV510 (30-F11, #9066967; BDBiosciences), anti-CD11b-PercP Cy5.5 (M1/10, #550993; BDBiosciences), anti-F4/50-APC Cy7(BM8, #123118; BioLegend), anti-Ly6G-PE-Cy7 (1A8, #560601; BDBiosciences), anti-SiglecF-APC (S17007L, #155507; BioLegend), anti-FcER1α−PE (REA1079, #130-118-896; Miltenyi), anti-CD49b-PercP Cy5.5 (HMα2, #103519; BioLegend), anti-CD3^−^ PercP Cy5.5 (145-2C11, #551163; BDBiosciences), anti-CD8-BV510 (H35-17.2, #740155; BDBiosciences), anti-CD4-PE-Cy7 (RM4-5, #552775; BDBiosciences), anti-CD45-B220-AF647 (RA3-6B2, #10229; BioLegend), anti-NK1.1-APC-H7 (PK136, #560618; BDBiosciences), anti-CD19-APC-H7 (1D3, #560143; BDBiosciences), anti-CD11c-APC (N418, #17-0114-81; eBioscience), and anti-MHC-II-Alexa700 (M5/114.15.2, #107621; BioLegend). Data were acquired on an LSRII (BD) and analyzed using FlowJo (Treestar) and Prism (GraphPad) software.

### TB staining for light microscopy

Mouse tissue samples (back and ear skin, tongue, heart, lungs, spleen, esophagus, duodenum, ileum, jejunum, and colon) were fixed in 10% formalin and embedded in paraffin. Mesenteric windows were collected according to standard procedures, fixed for 60 min in a 60% ethanol 70/20% chloroform/20% glacial acetic acid medium, and air dried. 4-μm-thick tissue sections and mesenteric windows were stained with TB according to standard procedures. Slides were scanned using a PANNORAMIC Digital Slide Scanner (3DHISTECH). The images acquired were analyzed using the slide viewing application CaseViewer 2.4.

### Immunostaining for confocal microscopy

A second set of mouse tissue samples (back and ear skin, tongue, lungs, trachea, esophagus, duodenum, ileum, jejunum, and colon) was embedded in tissue freezing medium (Scigen Tissue Plus OCT compound), snap-frozen, and sectioned at 25 µm on a cryostat (Leica). The GI tract (ileum, jejunum, and colon) was flushed with ice-cold PBS to remove luminal contents. Each part of the intestine was cut open longitudinally to perform Swiss rolls embedded in tissue freezing medium (Scigen Tissue Plus OCT compound), snap-frozen, and sectioned at 25 µm on a cryostat (Leica). The remaining parts of the intestine that were cut open longitudinally were stretched in a Sylgard plate. The muscularis externa was carefully removed from the remaining submucosa and lamina propria by gently scraping with forceps and fixed for 20 min in 4% paraformaldehyde. Immunostaining was performed according to standard procedures. Sections and intestinal whole-mount muscularis externa were permeabilized for 30 min in PBS supplemented with 0.5% BSA and 0.1% saponin. Permeabilized tissues were incubated overnight at 4°C with primary antibodies (anti-CD31-AF594 [WM59, #303126; BioLegend], anti-Tubb3-647 [TUJ1, #801209; BioLegend], anti-CD45-AF647 [30-F11, #103124; BioLegend], anti-MCPT1 [RF6.1, #14-5503-82; eBioscience], anti-GFP [PABG1, #PABG1; Chromotek], and anti-HuC/HuD [16A11, #A-21271; Thermo Fisher Scientific]), extensively washed, and incubated with appropriate secondary antibodies and/or Avidin (-Sulforhodamine, SRho, or -AF488) for 2 h at room temperature in the dark. Images of 1,024 × 1,024 pixels were acquired using a Zeiss LSM710 or a Leica TCS SP8 MP Meta inverted confocal laser-scanning microscopes. Images were processed using Zen software (Zeiss). Final image processing was done using Imaris software (Bitplane). Modeling and analysis of fluorescent signals were performed using untreated image sequences, as previously described (Gaudenzio et al., 2016), using Imaris software (Bitplane).

### Enzyme-linked immunosorbent assays (ELISAs) for total serum IgE, peanut-specific IgE, peanut-specific IgG1, and Mcpt-1 levels

An ELISA for total serum IgE was performed according to the manufacturer’s instructions using a Mouse IgE ELISA kit (#88-50460-88; Invitrogen); serum was applied at a concentration of 1:50. To measure peanut-specific IgE, a clear flat-bottom immuno nonsterile 96-well plate (#442404; Thermo Fisher Scientific) was coated with peanut extract in PBS (10 µg/ml) instead of the anti-IgE capture antibody, the rest of the assay was performed using Mouse IgE ELISA kit (#88-50460-88; Invitrogen) according to the manufacturer’s instructions; serum was applied at a concentration of 1:50. To measure peanut-specific IgG1, a clear flat-bottom immuno nonsterile 96-well plate (#442404; Thermo Fisher Scientific) was coated with peanut extract in PBS (10 µg/ml) instead of the anti-IgG1 capture antibody and the rest of the assay was performed using a Mouse IgG1 ELISA kit (#88-50410-88; Invitrogen), according to the manufacturer’s instructions; serum was applied at 1:5,000. Serum MCPT-1 levels were measured using an MCPT-1 (mMCP-1) Mouse ELISA Kit (#88-7503-88; Invitrogen) according to the manufacturer’s instructions; serum was applied at a concentration of 1:50.

### Peritoneal MC isolation and preparation for scRNAseq

Peritoneal lavages were performed by i.p. injection of 5 ml PBS 0.5 mM EDTA buffer and the resulting peritoneal fluid. Cells were incubated for 30 min with LIVE DEAD Cell Stain (Thermo Fisher Scientific) to determine viability. After lavage in PBS 10% FCS 2 mM EDTA, Fc receptors were blocked with an anti-mouse CD16/32 (S17011E, #156604; BioLegend) for 15 min at 4°C cells and then stained with anti-mouse CD45-APC (30-F11, #MCD4505; Thermo Fisher Scientific), anti-mouse-c-Kit-SB436 (ACK2, #62-1172-82; Thermo Fisher Scientific), and anti-mouse-IgE-AF488 (RME-1, #406910; BioLegend) antibodies. Negative lineage (Lin^−^) was a cocktail of anti-CD19-PE-Dazzle 594 (6D5, #115553; BioLegend), anti-Ly6G/C-PE- PE-Dazzle 594 (RB6-8C5, #108451; BioLegend), and an anti-CD11b-PE-CF594 (M1/70, #101255; BioLegend). MCs were sorted as LIVE DEAD^−^ CD45^+^ Lin^−^ c-Kit^+^ cells on FACS ARIA III and SORP instruments (BD) directly in refrigerated DMEM 10% FBS.

### RNAseq

Single-cell libraries were generated using the GemCode Single Cell Instrument and Single Cell 3′ Library & Gel Bead Kit v3 and B Chip Kit (10X Genomics) according to the manufacturer’s protocol. Briefly, after generation of nanoliter-scale gel bead-in-emulsions (GEMs), GEMs were reverse transcribed in a C1000 Touch Thermal Cycler (Bio-Rad), programmed at 53°C for 45 min, 85°C for 5 min, and held at 4°C. After reverse transcription, single-cell droplets were broken and cDNA was isolated and cleaned with Cleanup Mix containing DynaBeads (Thermo Fisher Scientific). cDNA was then amplified with a C1000 Touch Thermal Cycler programed at 98°C for 3 min, 12 cycles of (98°C for 15 s, 63°C for 20 s, 72°C for 1 min), 72°C for 1 min, and held at 4°C. Subsequently, the amplified cDNA was fragmented, end-repaired, A-tailed, index adaptor–ligated, and cleaned with cleanup mix containing SPRIselect Reagent Kit (Beckman Coulter) in between steps. Postligation product was amplified and indexed with a C1000 Touch Thermal Cycler programmed at 98°C for 45 s, 16 cycles of (98°C for 20 s, 54°C for 30 s, 72°C for 20 s), 72°C for 1 min, and held at 4°C. The sequencing-ready library was cleaned up with SPRIselect beads. All 10X libraries were charged at 1 nM with 1% PhiX and sequenced on NovaSeq6000 instrument (Illumina) with the following sequencing parameters: 28 bp read 1–8 bp index 1 (i7)–150 bp read 2. We used three SP lanes for a total of 388,074,304 reads (Perit. Digest: 87,477,668; Gut mucosa: 79,622,170; Skin: 220,974,466).

### Quality control numbers, ambient RNA decontamination, and analyses of scRNAseq samples

Cell Ranger software (v.7.0.1; 10X Genomics) was used to demultiplex Illumina BCL files to FASTQ files (CellRanger mkfastq), to perform alignment (to mouse GRCm38/mm10 genome), filtering, UMI counting, and to produce gene–barcode matrices (CellRanger count). Unless otherwise specified, all plots were generated by the Seurat visualization tools and by the ggplot2 library.

Counts matrices from CellRanger were processed with Seurat packages (version 4.1.3; Aran et al., 2019) in R (version 4.2.2). Ambient RNA contamination was estimated with the DecontX function from celda R packages (Yang et al., 2020) with default parameters, and then decontaminated count matrix was added as a new and default assay in the Seurat object. Dead cells and multiplets (i.e., droplets with more than one encapsulated cell) were identified based on quality metrics (minimum and maximum number of unique genes expressed and percentage of mitochondrial genes per cell). A threshold value for each variable was then determined for each dataset. For the peritoneal cavity dataset, retained cells were defined as cells having counts of >100 unique genes, <1,500 unique genes, and <10% of mitochondrial genes; there were 3,372 cells that met these criteria. For the skin dataset, retained cells were defined as cells having counts of >100 and <1,500 unique genes and <10% of mitochondrial genes; there were 7,166 cells that met these criteria. Finally, for the gut mucosa dataset, retained cells were defined as cells having counts of >100 and <3,000 unique genes and <10% of mitochondrial genes; there were 2,121 cells that met these criteria. The second step consisted in running the Seurat pipeline: raw counts were log-normalized with NormalizeData() function, then variable features were determined with FindVariableFeatures(), and normalized counts were then scaled on the list of variable features using the ScaleData() function. PCA was performed with RunPCA() with default parameters. For dimension reduction and visualization, UMAP coordinates were calculated in the PCA space by using the implemented function RunUMAP() on all 50 computed PCs.

### Identification of cells and calculation of cell type similarity score

Cell identification was done using the celldex database (version 1.0.0) and SingleR (version 1.4.0) in R packages (Aran et al., 2019). Based on the celldex database, SingleR is able to compute a pairwise-correlation study based on Spearman ranked correlation, resulting in the generation of a cell similarity score. Using SingleR, similarity scores were assigned in accord with the main immune cell profiles from ImmGen database (B cells, T cells, dendritic cells, macrophages, MCs, monocytes, natural killer cells, and T cells) for each of the cells included in the scRNAseq. The identity of a given cell in the dataset was then given by the higher similarity score obtained using singleR.

### scRNAseq integration workflow

Two integrated datasets were built for this study: first, with only in-house experiment and last integrating in-house data with datasets from Mouse Cell Atlas and GSE122930 for heart dataset. Both were integrated following Seurat reciprocal PCA integration workflow: (1) all data were loaded independently, (2) features for integration were selected with SelectIntegrationFeatures() function, (3) dataset where scaled and PCA were computed based on previously selected features applying ScaleData() and RunPCA() function on each dataset, (4) integration anchors were defined using FindIntegrationAnchors(), (5) finally, data were integrated using IntegrateData() and the previously defined anchors, with default parameters. Once whole data were integrated, MCs were isolated based on previous identification.

### Data integration and pseudo-bulk profiling

Using the first integrated object, containing only our in-house generated three datasets, and to minimize the weight of the high number of MCs in the peritoneal dataset (defined as MCs in the Fig. 1 A), we did a random subsampling of 50 cells. To study the impact of this subsampling, twenty random samplings were done and then the DEGs were analyzed. Results showed that MCs from the peritoneal/digestive cavity were homogeneous and the 20 sampled datasets expressed canonical MC markers. DEGs between *Mrgprb2*^*+*^ and *Mcpt1*^*+*^ MCs, executed using the FindMarkers() function, returned a list of 187 genes (only 74 were shown) with false dicovery rate <0.05 and limit fold change ≥0.25, including *Mrgprb2* and *Mcpt1*. We showed these differences by plotting a density plot based on gene-weighted kernel density estimation realized with Nebulosa packages (v1.8.0; Alquicira-Hernandez and Powell, 2021) and by doing a pseudo-bulk PCA between expression profiles. Pseudo-bulk data were generated with the AverageExpression() function and PCA was based on the top 500 variable features.

### Global analysis of mice MCs

MCs were screened and isolated from six datasets from adult intestine, adult stomach, neonatal skeletal muscle, neonatal skin, neonatal uterus, and mammary gland of pregnant mice in the Mouse Cell Atlas. Other datasets were also used for the control heart (GSE122930). All these datasets were cleaned from ambient RNA contamination using DecontX and processed following the standard Seurat pipeline. Although data from Mouse Cell Atlas are already identified, we checked the presence of MCs using SingleR and Immgen databases. All selected datasets from Mouse Cell Atlas and heart were integrated with our scRNAseq data (with subsampled peritoneal cavity data) as described previously and MCs were isolated based on previous identification. UMAP coordinates were calculated with RunUMAP function based on 30 first PCs, and density plots showing expression of *Mrgprb2* and *Mcpt1* were realized with Nebulosa R packages. We defined two groups: Mrgprb2^+^/Mcpt1^−^ and Mrgprb2^−^/Mcpt1^+^, and a DEGs study between these two groups was computed with FindAllMarkers() function based on Wilcoxon’s test and found 322 genes (only 85 were shown) with false discovery rate <0.05 and limit fold change ≥0.25. Finally, we computed an enrichment score using AddModuleScore() function based on an already known list of genes: genes with 10-fold higher expression levels in MCs described in Dwyer et al. (2016) and top 50 DEGs between B7high and B7low described in Derakhshan et al. (2021).

### Analysis of human datasets from CZ BioHub Tabula Sapiens

Human data came from CZ BioHub Tabula Sapiens (Tabula Sapiens Consortium et al., 2022). As stated in their publication, “donated organs and tissues were procured at various hospital locations in the Northern California region through collaboration with a not-for-profit organization, Donor Network West (DNW). DNW is a federally mandated organ procurement organization for Northern California. Recovery of non-transplantable organs and tissues was considered for research studies only after obtaining records of first-person authorization (i.e., donor’s consent during his/her DMV registrations) and/or consent from the family members of the donor. Single-cell suspensions from each organ were prepared in tissue expert laboratories at Stanford and UCSF. For some tissues, the dissociated cells were purified into compartment-level batches (immune, stromal, epithelial, and endothelial) and then recombined into balanced cell suspensions to enhance sensitivity for rare cell types. The research protocol was approved by the DNW’s internal ethics committee (Research project STAN-19-104) and the medical advisory board, as well as by the Institutional Review Board at Stanford University which determined that this project does not meet the definition of human subject research as defined in federal regulations 45 CFR 46.102 or 21 CFR 50.3”.

Data are already preprocessed and cleaned from dead cells, multiplet, or ambient RNA as described in the original publication (Tabula Sapiens Consortium et al., 2022). Data were processed with the classical Seurat pipeline. Cells identified by CZ BioHub as MCs and grouped within cluster 31 were selected as MCs. The selection was confirmed by studying the expression of canonical markers CPA3, KIT, FCER1A, and TPSB2. Tissue represented by <10 cells were removed from the dataset (eye, fat, prostate, and thymus). As isolated mast cells came from donors of different gender and were sequenced with different technologies (85% from 10X Genomics and 15% from SmartSeq2), we first performed a regression of the dataset during the scaling step using the “var.to.regress” parameter taking into account both gender and method. We then applied another Harmony integration step on the gender to correct for eventual gender driven bias.

As CTMC/MMC dichotomy in humans is not as simple as in the mouse model, selected MCs were projected in their own UMAP space and then clustered with the FindNeighbors() and FindClusters() functions.

We applied pseudo-bulk transformation with the AverageExpression() function and realized a hierarchical clustering based on the correlation distance. The resulting dendrogram allowed us to define six MC clusters in the human dataset. DEGs studies were conducted between these six clusters and between organ tissues within each MC cluster. Another enrichment score was computed using AddModuleScore() function based on all the DEGs characterizing MC1, MC3, and MC4 population described by Dwyer et al. (2021) (no DEGs were found to specifically characterized the MC2).

### Cell count

MCs were counted on either TB-stained or anti-Mcpt1-FITC (RF6.1, #14-5503-82; eBioscience) and Avidin (Av.SRho or Av.488)-stained tissue sections, and macrophages were counted on tissue sections stained with anti-CD45-AF647 (30-F11, #103124; BioLegend) and anti-CX3CR1-BV650 (SA011F11, #149033; BioLegend) from *Mrgprb2-Cre; iDTR*^*fl/fl*^ and their littermate controls previously treated with dt. Avidin^+^ gut MCs (gMCs) that are rare were counted manually, whereas all other cells were counted automatically using Imaris software (Bitplane). Briefly, fluorescence corresponding to the marker of interest (Mcpt1, CD45, or CX3CR1) was modeled into matched 3D objects using the isosurface algorithm, and the number of surfaces was counted. All sections were “coded” so the evaluator was not aware of their identity, as previously described.

### ELISAs

An ELISA for total serum IgE was performed according to the manufacturer’s instructions using a Mouse IgE ELISA kit (#88-50460-88; Invitrogen); serum was applied at a concentration of 1:50. To measure peanut-specific IgE, a clear flat-bottom immuno nonsterile 96-well plate (#442404; Thermo Fisher Scientific) was coated with peanut extract in PBS (10 µg/ml) instead of the anti-IgE capture antibody, and the rest of the assay was performed using Mouse IgE ELISA kit (#88-50460-88; Invitrogen) according to the manufacturer’s instructions; serum was applied at a concentration of 1:50. To measure peanut-specific IgG1, a clear flat-bottom immuno nonsterile 96-well plate (#442404; Thermo Fisher Scientific) was coated with peanut extract in PBS (10 µg/ml) instead of the anti-IgG1 capture antibody, the rest of the assay was performed using a Mouse IgG1 ELISA kit (#88-50410-88; Invitrogen), according to the manufacturer’s instructions; serum was applied at 1:5,000. Serum MCPT-1 levels were measured using an MCPT-1 (mMCP-1) Mouse ELISA Kit (#88-7503-88; Invitrogen) according to the manufacturer’s instructions; serum was applied at a concentration of 1:50.

### Statistics

Statistical tests were performed with the software Prism 8 (GraphPad Software). Two-tailed unpaired/paired Student’s *t* tests, one-way ANOVA with Tukey’s test for multiple comparisons, or Mann–Whitney test was performed on samples as noted in the respective figure legends. A P value of <0.05 was considered statistically significant.

### Online supplemental material

Fig. S1 shows the specificity of mRNA expression of *MrgprB2* in MCs among immune cells and DRG neurons. Fig. S2 shows the specific expression of Tdt reporter in connective tissue MCs of *MrgprB2-cre*; *Tdt*^*fl/fl*^ mice. Fig. S3 shows the trajectories analysis suggesting that MrgprB2^high^ MCs in the skin represent a mature population of MCs in the mouse. Fig. S4 shows the homeostatic renewal of MrgprB2^+^ and Mcpt1^+^ MCs in the intestine and the absence of Tdt expression in MrgprB2^+^ and Mcpt1^+^ MCs in the skin and intestine of *Ms4a3-cre*^*+*^*; tdTomato* mice. Fig. S5 shows that selective depletion of MrgprB2^+^ MCs across organs does not impact other immune cells. Table S1 shows DEGs between CTMCs and MMCs populations in mouse datasets. Table S2 lists tissue imprinting DEGs within CTMCs and MMCs. Table S3 shows DEGs between all the six characterized MC populations in human datasets. Table S4 shows genes selected as signatures for each group of characterized MC populations in human datasets. Table S5 shows genes selected for tissue imprinting score.

## Supplementary Material

Table S1 shows DEGs between CTMCs and MMCs populations in mouse datasets.

Table S2 lists tissue imprinting DEGs within CTMCs and MMCs.

Table S3 shows DEGs between all the six characterized MC clusters in human datasets.

Table S4 shows genes selected as signatures for each group of characterized MC clusters in human datasets.

Table S5 shows genes selected for tissue imprinting score.

## Data Availability

Raw and processed data are available in the nonprofit repository Dryad (https://doi.org/10.5061/dryad.np5hqbzzz). All data needed to evaluate the conclusions in the paper are present in the article and/or the supplementary material.
