# Correction: Landscape of mast cell populations across organs in mice and humans

**DOI:** 10.1084/jem.2023057001172024c

**Published:** 2024-01-24

**Authors:** Marie Tauber, Lilian Basso, Jeremy Martin, Luciana Bostan, Marlene Magalhaes Pinto, Guilhem R. Thierry, Raïssa Houmadi, Nadine Serhan, Alexia Loste, Camille Blériot, Jasper B.J. Kamphuis, Mirjana Grujic, Lena Kjellén, Gunnar Pejler, Carle Paul, Xinzhong Dong, Stephen J. Galli, Laurent L. Reber, Florent Ginhoux, Marc Bajenoff, Rebecca Gentek, Nicolas Gaudenzio

Vol. 220, No. 10 | https://doi.org/10.1084/jem.20230570 | July 18, 2023

After publication of the article, some readers reached out to *JEM* to raise concerns about Figs. 5 and 6, specifically regarding the clustering of human mast cells. They highlighted that the analysis did not correct for the influence of donors’ gender and/or sequencing technique on the conclusions. At *JEM*’s request, the authors reanalyzed the data to correct for such bias and identified six different mast cell clusters/states with distinct transcriptomic signatures across twelve organs in humans (the previous analysis had identified seven mast cell clusters/states). Accordingly, the clustering of human mast cells and associated mast cell signatures have been revised in the text, in Figs. 5 and 6, and in Tables S3–S5. The conclusions regarding the heterogeneity and diversity of human mast cells remain unchanged.

The corrected Figs. 5 and 6 are shown here, as well as changes to the text and figure legends (indicated in bold). Tables S3–S5 have been replaced online. The errors appear in print and PDFs downloaded before January 17, 2024.

## Abstract

Mast cells (MCs) are tissue-resident immune cells that exhibit homeostatic and neuron-associated functions. Here, we combined whole-tissue imaging and single-cell RNA sequencing datasets to generate a pan-organ analysis of MCs in mice and humans at steady state. In mice, we identify two mutually exclusive MC populations, MrgprB2^+^ connective tissue–type MCs and MrgprB2^neg^ mucosal-type MCs, with specific transcriptomic core signatures. While MrgprB2^+^ MCs develop in utero independently of the bone marrow, MrgprB2^neg^ MCs develop after birth and are renewed by bone marrow progenitors. In humans, we unbiasedly identify **six** MC **clusters/states** (**MC1–6**) distributed across 12 organs with different transcriptomic core signatures. **MC1 are preferentially enriched in the skin and lungs, MC2, MC3, and MC4 in the skin and bladder, MC5 in the lymph node and vasculature, and MC6 in the trachea and lungs.** This comprehensive analysis offers valuable insights into the natural diversity of MC subtypes in both mice and humans.

## Introduction ***(fourth paragraph)***

Here, we integrate multiple single-cell datasets from in-house and publicly available sources (all decontaminated from potential ambient tissue mRNA) to build a comprehensive overview of mouse and human MC populations across organs. In the mouse, CTMCs expressing MrgprB2^+^ have a common gene expression program across organs, such as in skin, muscle, uterus, mammary gland, peritoneal cavity, and heart. By contrast, MrgprB2^neg^ MMCs are transcriptionally distinct from MrgprB2^+^ CTMCs and are mostly found in the digestive tract. While MrgprB2^+^ CTMCs develop during embryogenesis and are independent of the BM for renewal, MrgprB2^neg^ MMCs arise postnatally, require signals from the microbiota, and are renewed by Ms4a3^neg^ BM progenitors. We found that MrgprB2^+^, but not MrgprB2^neg^, MCs are required for food-induced systemic anaphylaxis. In humans, the unbiased analysis of all MCs identified across 24 organs in the human cell atlas revealed the presence of **six** distinct populations/states, named **MC1–6**, with specific groups of genes allowing their precise identification. **We show that the six MC clusters can be found heterogeneously distributed across the skin, lung, pancreas, skeletal muscle, tongue, bladder, large and small intestines, lymph nodes, mammary glands, trachea, and vasculature, with nevertheless organ-specific enrichments per clusters.**

## Results

### Integrated single-cell analysis of human organs identifies six distinct MC states

Of relevance for drug development and MC-related therapies, we next investigated the transcriptomic heterogeneity of human MC populations across different organs. We aggregated the databases of 24 different organs from the Tabula Sapiens, which is already decontaminated from ambient RNA with Decontx (Yang et al., 2020), as part of the processing guidelines (https://tabula-sapiens-portal.ds.czbiohub.org/), in a unique UMAP composed of 264,009 single cells (Fig. 5 A). **Taking into account differences in gender and sequencing technologies, we applied two additional layers of integration (detailed in the Materials and methods section) to avoid any potential bias of analysis.** We next identified a unique cluster composed of 2,690 MCs based on the signature of the cardinal MC genes *KIT*, *CPA3*, *TPSB2*, and *CMA1* (Fig. 5, B and C). We then projected all of the identified MCs on the same UMAP, reaching a total number of 2,690 single MCs from 12 different organs. The human datasets appear to be more complex and heterogeneous than the mouse datasets, and we could not observe an obvious CTMC/MMC transcriptomic dichotomy in humans based on the expression of the classical histochemical markers *CMA1* and *TPSB2* reported in the literature (Derakhshan et al., 2022).

We, therefore, decided to adopt an unbiased approach to better understand human MC heterogeneity and performed an unsupervised nearest-neighbor analysis that identified the presence of **six** potential clusters (Fig. 5 D). To decipher the number of MC subsets present among these **six** clusters, we generated a correlation heatmap (Fig. 5 E) followed by a cluster dendrogram (Fig. 5 F) to directly visualize the strength of relationships between the different clusters. We could **confirm** the presence of **six** potential MCs **clusters** (Fig. 5 G) with a total of **1,570** statistically significant DEGs that defined the transcriptomic heterogeneity between each **cluster** of MCs (Fig. 5 H). The total list of DEGs characteristic to each of the human MC **clusters**, hereafter named **MC1–6**, is provided in Table S3.

Among the many DEGs, MC1 notably **expressed genes encoding the TNF superfamily member 12 (TNFSF12), the leukotriene C4 synthase (LTC4S), the suppressor of cytokine signaling 1 (SOCS1), and the GRB2-related adaptor protein 2 (GRAP2).** MC2 notably **were characterized by the expression of genes encoding interleukin 13 (IL13), the pleckstrin homology-like domain family A member 1 (PHLDA1), the early growth response 3 gene (EGR3), prostaglandin-endoperoxide synthase 2 (PTGS2), and the cluster differentiation 83 (CD83).** MC3 expressed, among others, the **cathepsin G (CTSG), the gene encoding the neuropeptide PACAP (ADCYAP1), and the chemokine CCL2.** MC4 expressed genes encoding **growth factors (VEGFA, CSF1), the glucocorticoid receptor (NR3C1), and cytokine receptors (IFNGR1, IL18R1).** MC5 was preferentially enriched in genes encoding the **chymase 1 (CMA1), the chemokine receptor CXCR4, the cathepsin G (CTSG), IL5 receptor alpha (IL5RA), and multiple members of the nuclear receptor subfamily 4 group A (NR4A1/2/3). Finally, MC6 was preferentially enriched in genes encoding integrins (IGAM, ITGB2), neuronal growth factor receptor (NTRK1), and the common unit of IL3 and IL5 receptors (CSF2RB).** Interestingly, *MRGPRX2* was found heterogeneously expressed among clusters with a tendency for enrichment in **MC5 and MC2** but, *MRGPRX1*, another receptor reported to be the human ortholog of *Mrgprb2*, could not be found in any dataset.

We then isolated each MC’s DEGs signature (Table S4) **and isolated a reduced list of 10 genes to establish a “core signature” that would identify each MC state. We then projected the score of each of these “core signatures”** on the aggregated UMAP of MCs (Fig. 5 I). Using this approach, we could confirm that each identified set of DEGs enabled the precise identification of the corresponding MCs in the UMAP.

Finally, we extracted scRNAseq transcriptomic signatures from the states of MCs identified in chronic rhinosinusitis with nasal polyposis patients by Dwyer et al. (2021) and projected them on our aggregated UMAP of MCs (Fig. 5 J). We could show that the **MC_3** populations identified by Dwyer et al. (2021) matched several of our populations whereas **their MC1 signature matched two discrete MC clusters in our datasets, namely MC2 and MC5 clusters.** These data demonstrate that at least **six** MC **clusters/states** with distinct transcriptomic signatures exist across organs in humans and strongly suggest that the heterogeneity of human MCs might extend far beyond the classical CTMC/MMC dichotomy.

### Distribution of the six MC states across organs in human datasets

We then investigated the anatomical distribution of the **six** MC **clusters** in the different human organs (Fig. 6 A). We found that **the six MC clusters were heterogeneously distributed among different organs, but some organs displayed restricted representations of MC states, such as the large and small intestines (MC2 and MC5 only), lymph node (majority of MC5), and lung (MC1 and MC6)** (Fig. 6 B).

The distribution pattern of the different MC states prompted us to investigate potential common makers among them. We first investigated whether different MC subsets could express common gene expression features when located in the same tissue. We analyzed the common gene expression pattern of **MC1, MC3, and MC4** to identify DEGs previously reported to be important players in skin homeostasis (Fig. 6 C). We created an enrichment score (the list of genes is provided in Table S5) that could reflect a potential skin-related biological process and projected it on the aggregated UMAP of all MCs population. We found that such a genes signature was restricted to those MCs found in the skin but not in other organs (Fig. 6 D), among which were *ADM*, *RARA*, and *LAMA5*, which encode key components of skin homeostasis (Meixiong et al., 2019; Szymański et al., 2020; Wegner et al., 2016; Fig. 6 E).

Because the lungs were found to be composed of **MC1 and MC6 clusters**, we, therefore, investigated the presence of DEGs previously reported to be involved in lung homeostasis (Fig. 6 F). We created a lung MC enrichment score (Table S5) and projected it on the aggregated UMAP representing all MC populations. We found a common signature of genes among the three MC subsets that was restricted to those MCs found exclusively in the lungs (Fig. 6 G), among which were *APOE* and *MATK* (megakaryocyte-associated tyrosine kinase), both previously reported to play a role in lung diseases (Gordon et al., 2019; Xiao et al., 2012; Zhong et al., 2021; Fig. 6 H). We performed the same experiment with the bladder, which is composed of **MC2, MC3, MC4, and MC6** (Fig. 6 I and Table S5). In such MC states, we notably found a common signature of genes restricted to the bladder (Fig. 6 J), among which are *IL13* and *PHLDA1* (Fig. 6 K). These data strongly suggest that different MC states found in the same organ could share the expression of common genes involved in the homeostasis of the tissue in which they reside.

## Discussion

### Heterogeneity and distribution of MCs in different anatomical locations in mice and humans ***(fourth and fifth paragraphs)***

We used the large publicly available dataset from the Tabula Sapiens consortium to generate an aggregated UMAP composed of cells from 24 human organs. From this UMAP, we used the canonical MC markers *KIT*, *CPA3*, *TPSB2*, and *CMA1* to extract the cluster of MCs. When we analyzed the expression profile of *TPSB2* and *CMA1*, we could not find a clear CTMC (i.e., positive for *TPSB2* and *CMA1* or *MRGPRX2*) versus MMC (i.e., positive only for *TPSB2*; at least according to previous literature based on histochemical analyses). We, therefore, decided to take an unbiased approach and identified the presence of **six** distinct MCs clusters based on the presence of many DEGs between these clusters (with **MC2 and MC3** being relatively close to each other). Importantly, we mentioned the word **“cluster” or “state”** to qualify the distinct MC transcriptomic profiles identified, as it is still unclear whether they represent different transcriptomic states of MCs or truly different MC subsets with different origins and/or renewal dynamics.

It thus appears that the complexity and transcriptomic heterogeneity of human MCs goes beyond the classical CTMC/MMC dichotomy that we observed in mice, and that, among what have been called CTMCs or MMCs in humans, there should be different varieties of MCs with distinct transcriptomic programs. Among the **six** different human MC **clusters/states** that we identified in this study, **they do not seem to be restricted to a single organ.** Our classification also adds a layer of complexity to the previous study that identified four different populations of MCs by scRNAseq in polyps from patients suffering from chronic rhinosinusitis (Dwyer et al., 2021).

### Differential origin and renewal dynamics of MCs ***(last paragraph)***

In conclusion, our study provides a new perspective on the heterogeneity and tissue-specific specialization of MCs both in the mouse and in humans. Altogether, we reveal an unexpected level of transcriptomic heterogeneity, particularly among the human MC populations. Our data support the existence of at least **six distinct transcriptionally defined MC clusters** in humans in addition to the two main MC populations (i.e., CTMCs and MMCs) in mice. Approaches such as those we have used in this study should be applied to data derived from even more anatomical locations in both mice and humans, and the results may be helpful in defining additional distinct subtypes of MCs in both species. The extent to which and how this can influence organ-level functions and immune responses remain to be investigated. In aggregate, this study constitutes an online resource that regroups multiple MC single-cell transcriptomic profiles from various mouse and human organs and should help to refine MCs annotation and better understand their specialized functions across organs.

## Materials and methods

### Analysis of human datasets from CZ BioHub Tabula Sapiens ***(second and fourth paragraphs)***

Data are already preprocessed and cleaned from dead cells, multiplet, or ambient RNA as described in the original publication (Tabula Sapiens Consortium et al., 2022). Data were processed with the classical Seurat pipeline. Cells identified by CZ BioHub as MCs and grouped within cluster 31 were selected as MCs. The selection was confirmed by studying the expression of canonical markers CPA3, KIT, FCER1A, and TPSB2. Tissue represented by <10 cells were removed from the dataset (eye, fat, prostate, and thymus). **As isolated mast cells came from donors of different gender and were sequenced with different technologies (85% from 10X Genomics and 15% from SmartSeq2), we first performed a regression of the dataset during the scaling step using the “var.to.regress” parameter taking into account both gender and method. We then applied another Harmony integration step on the gender to correct for eventual gender driven bias.**

We applied pseudo-bulk transformation with the AverageExpression() function and realized a hierarchical clustering based on the correlation distance. The resulting dendrogram allowed us to define **six** MC clusters in the human dataset. DEGs studies were conducted between these **six** clusters and between organ tissues within each MC cluster. Another enrichment score was computed using AddModuleScore() function based on all the DEGs characterizing MC1, MC3, and MC4 population described by Dwyer et al. (2021) (no DEGs were found to specifically characterized the MC2).

**Figure 5.**
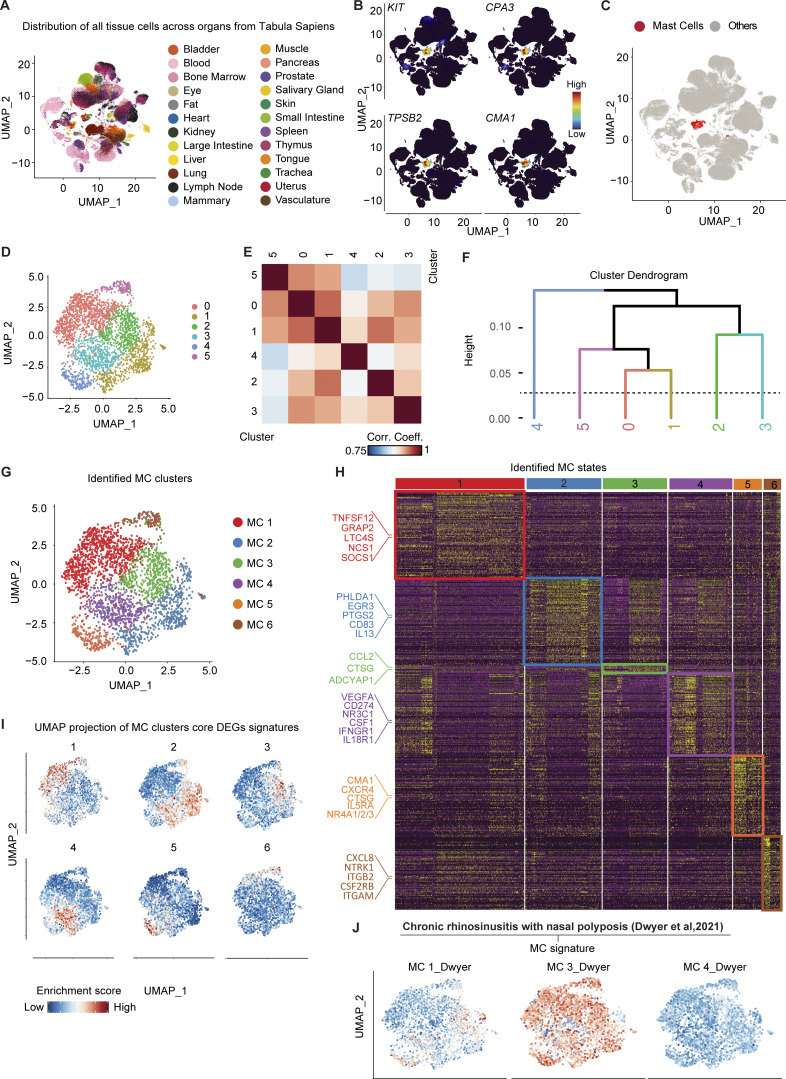
**Transcriptomic profiling of human MCs across tissues. (A)** UMAP plot of the distribution of all tissue cells across organs from the Tabula Sapiens. **(B)** UMAP plot of the expression density of CPA3, KIT, TPSB2, and CMA1 on the cells from the Tabula Sapiens. **(C)** UMAP showing the final MC **clusters** selected for further clustering. **(D)** UMAP of the Louvain clustering of the selected MCs. **(E)** Heatmap of correlation between the **six** Louvain communities after pseudo-bulk transformation. **(F)** Hierarchical clustering of Louvain communities based on the distance of correlation. Populations were grouped if the distance between them was inferior to 0.1 (dotted line). **(G)** UMAP showing the final **six** states of human MCs identified. **(H)** Heatmap of 350 representative DEGs between the **six** populations of MCs. Genes of interest of each population are highlighted on the left. **(I)** UMAP showing the score-based identification of each of the **six** states using a selected set of markers. **(J)** UMAP showing the score-based identification of the **six** states of human MCs using the signature of MC1, MC2, and MC4 populations defined in Dwyer et al. (2021).

**Figure 6.**
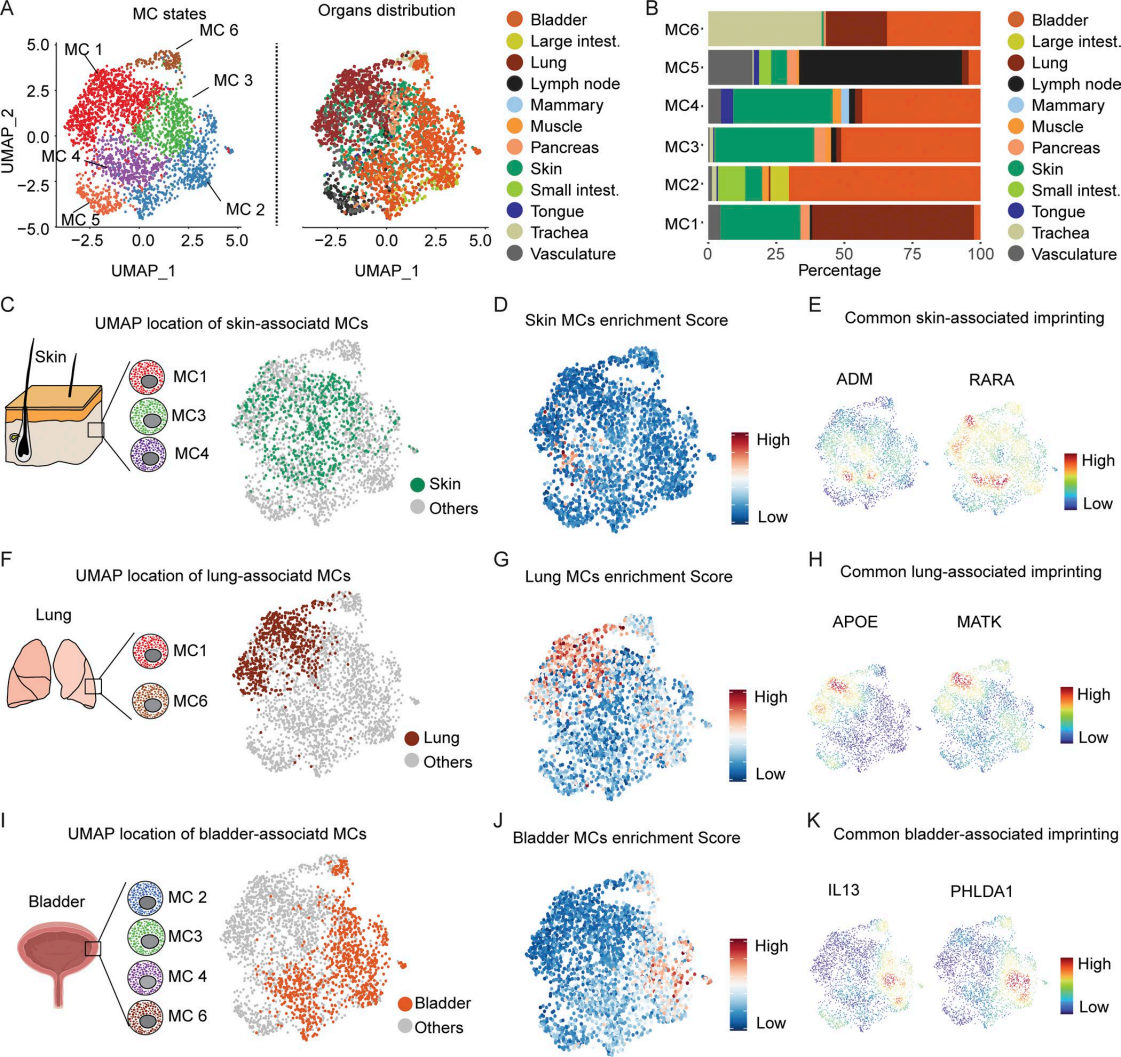
**Distribution of human MC states across organs and genes expression in major subsets from the same organ. (A)** UMAP representing the human MCs colored according to the **six** identified **clusters** (left UMAP) or the organ of origin (right UMAP). **(B)** Bar graph showing the proportion of each MC states in the different organs. **(C)** Picture showing the MC **cluster** present in the skin (left) and their distribution on the UMAP (right). **(D)** UMAP showing the score-based identification of skin MCs using a common set of markers from **MC1, MC3, and MC4** states. **(E)** Examples of UMAPs showing the expression density of common sets of skin-associated genes. **(F)** Picture showing the MCs present in the lung (left) and their distribution on the UMAP (right). **(G)** UMAP showing the score-based identification of lung MCs using a common set of markers from **MC1 and MC6** populations. **(H)** Examples of UMAPs showing the expression density of lung-associated genes. **(I)** Picture showing MC states present in the bladder (left) and their distribution on the UMAP (right). **(J)** UMAP showing the score-based identification of bladder MCs using a common set of markers from **MC2, MC3, MC4, and MC6** states. **(K)** Examples of UMAPs showing the expression density of bladder-associated genes.

